# A practical guide to long-term field PAM chlorophyll fluorescence measurements: setup, installation, data processing with R package ‘LongTermPAM’ and interpretation

**DOI:** 10.1007/s11120-025-01166-1

**Published:** 2025-09-04

**Authors:** Chao Zhang, Erhard E. Pfündel, Jon Atherton, Juho Aalto, Jia Bai, Toivo Pohja, Paulina A. Rajewicz, Albert Porcar-Castell

**Affiliations:** 1 Optics of Photosynthesis Laboratory, Institute for Atmospheric and Earth System Research (INAR)/Forest Sciences, Viikki Plant Science Centre (ViPS), University of Helsinki, Helsinki, 00014 Finland; 2 Heinz Walz GmbH, Eichenring 6, D-91090 Effeltrich, Germany; 3 Institute for Atmospheric and Earth System Research (INAR)/Forest Sciences, University of Helsinki, Helsinki, 00014 Finland; 4 Hyytiälä Forest Station, Hyytiäläntie 124, Korkeakoski, 35500 Finland; 5 State Key Laboratory of Remote Sensing Science, Faculty of Geographical Science, Beijing Normal University, Beijing, 100875 China; 6 Beijing Engineering Research Center for Global Land Remote Sensing Products, Faculty of Geographical Science, Beijing Normal University, Beijing, 100875 China

**Keywords:** Monitoring PAM, Non-photochemical quenching, Norway spruce, Photochemical quenching, Seasonality of photosynthesis, Scots pine

## Abstract

Pulse-amplitude modulated (PAM) chlorophyll fluorescence (ChlF) measurements provide a non-invasive method to study the regulation of the light reactions of photosynthesis in situ. PAM ChlF contributes also to the advancement of the interpretation of long-term observations of remotely sensed solar induced fluorescence by revealing the mechanistic connection between ChlF and photosynthetic function. However, long-term field PAM ChlF measurements remain uncommon due to challenges associated with the outdoor environment, instrument installation and maintenance, or data processing and interpretation. We here provide guidelines and recommendations to support long-term field installation of PAM ChlF systems, including the design of specialized field installation supports. We also introduce a dedicated R-package (LongTermPAM) to help users filter and analyse long-term data. Methods are demonstrated using two long-term datasets obtained with a MONI-PAM system (Monitoring PAM, Walz GmbH, Germany) on Scots pine and Norway spruce in a boreal forest. The LongTermPAM R-package helped filter spurious observations caused by dew, ice or snow, permitting calculation of photochemical (PQ) and non-photochemical quenching (NPQ) parameters and their associated yields. Finally, we illustrate how PQ and NPQ regulate the relationship between ChlF and photochemical yields, and discuss how variations in leaf PAR absorption, energy partitioning between photosystems II and I, and the contribution of photosystem I to the total ChlF signal can influence the interpretation of PAM ChlF, emphasizing the value of complementary measurements to capture variability in these factors. Although based on our experience with the MONI-PAM system in a boreal environment, most issues hereby addressed can be broadly applied to other long-term PAM monitoring systems and environments.

**Supplementary Information:**

The online version contains supplementary material available at 10.1007/s11120-025-01166-1.

## Introduction

Chlorophyll-a fluorescence (ChlF) is electromagnetic radiation in the 650–850 nm range, emitted by the light harvesting antennae of photosystems nanoseconds after light absorption (Porcar-Castell et al. 2014). For decades, the intensity, kinetics, and spectral properties of the ChlF emission have helped elucidate the structural and functional regulation of photosynthesis (Govindjee 1995; Murchie and Lawson 2013). More recently, remote sensing of solar-induced chlorophyll fluorescence (SIF) provides opportunities to expand the study of photosynthesis to new domains both temporally (from diurnal to seasonal scales) and spatially (from leaves to ecosystems) (Porcar-Castell et al. 2021; Sun et al. 2023a). Yet, although the connection between ChlF and photosynthesis is relatively well understood at the diurnal scale, the mechanisms connecting ChlF and photosynthesis at the seasonal scale remain elusive, which limits our capacity to assimilate and interpret datasets obtained via remote sensing of SIF (Porcar-Castell et al. 2021; Sun et al. 2023b). In this context, field and long-term measurements of pulse amplitude modulated (PAM) fluorescence can contribute to elucidate the seasonal regulation of photosynthesis and advance the interpretation of long-term SIF datasets. For example, PAM fluorometers can resolve the dynamics of ChlF and photochemical yields at the leaf level, the smallest scale at which ChlF and photosynthesis can be measured in vivo; and for evergreen species, where the seasonal regulation of photosynthesis is most prominent (Kim et al. 2021; Martini et al. 2022; Pierrat et al. 2024; Rajewicz et al. 2023; Zhang et al. 2019).

Since their introduction four decades ago (Schreiber et al. 1986), PAM fluorometers have continued to expand the range of possibilities to study photosynthesis. In particular, the emergence of monitoring PAM systems for long-term, unattended field applications enables the study of the environmental regulation of photochemical (PQ) and non-photochemical (NPQ) quenching processes in leaves, in situ, and with unprecedented temporal resolution and coverage (Porcar-Castell 2011; Porcar-Castell et al. 2008a). The dynamics of PQ and NPQ are associated to the allocation of absorbed excitation energy to photochemistry and regulated thermal dissipation, respectively. These processes are addressed as quenching because, by consuming excitation energy, they reduce (i.e. quench) the emission of fluorescence, setting the basis for the use of ChlF to study PQ and NPQ dynamics (Maxwell and Johnson 2000). The growing interest in long-term in situ measurements of leaf-level PAM ChlF has spurred the development of new instrumentation, beyond the original Monitoring PAM system, such as the MICRO-PAM (Heinz Walz GmbH, Effeltrich, Germany), the Monitoring Pen MP-100 (Photon Systems Instruments, Brno, Czech Republic) or the Plant Stress Probe PSP-32 (Opti-Sciences, Hudson, U.S.A). Yet, despite their considerable informative potential, long-term PAM measurements under field conditions remain uncommon. Main obstacles for their implementation are challenges associated with the outdoor environment, and uncertainties related to the absence of guidelines for instrument setup, installation, maintenance, and the processing and interpretation of the resulting long-term data. For example, a first obstacle to the interpretation of long-term PAM ChlF values (hereafter PAM-F) recorded with monitoring instrumentation, was the absence of equations compatible with the estimation of both diurnal and seasonal quantum yields and quenching parameters derived from a continuous and long-term dataset. To this end, Porcar-Castell (2011) defined a set of diurnal and seasonal parameters and introduced the parameter *PQ* (Photochemical Quenching) for consistency with the widely used Stern-Volmer *NPQ* (Non-Photochemical Quenching) parameter (Bilger and Björkman 1990), which are further introduced in the following sections. Hereafter we use the italics *PQ*/*NPQ* when referring to the parameters derived from PAM-F, and the normal font when referring to the processes. The unfamiliar reader is encouraged to browse through some of the excellent overviews on PAM-F before moving on (Kalaji et al. 2017, 2014; Lazár 2015; Logan et al. 2007; Maxwell and Johnson 2000; Murchie and Lawson 2013) as well as the original paper by Porcar-Castell (2011).

This communication aims to provide a practical guide to support the deployment of long-term field PAM ChlF measurements, as well as the processing and interpretation of the resulting data. More specifically, we: (1) introduce guidelines and recommendations to facilitate the setup and installation of long-term PAM ChlF measurements under field conditions, including the design of specialized supports for mounting cylindrical-type fluorometers in woody vegetation; (2) present and demonstrate a dedicated R-package (LongTermPAM) for automatically removing artifactual data from PAM datasets and estimating ChlF parameters; and, (3) provide an overview of the theory and assumptions underlying long-term PAM measurements, demonstrating how PQ and NPQ shape the observed relationships between PAM-F levels and photochemical yields, and discussing both the potential and limitations of these measurements. The methods are demonstrated using two long-term datasets obtained with a Monitoring PAM system, hereafter referred to as MONI-PAM (Monitoring PAM, Walz GmbH, Germany), measuring in Scots pine and Norway spruce needles growing in a boreal forest. Finally, although the paper is based on our experience with the above system in a boreal environment, we try to be as generic as possible, hoping that the practical and theoretical aspects introduced in this communication can be broadly applicable to users of different instruments or in other environments.

## Guidelines for the implementation of long-term field PAM ChlF

### Measuring principles and assumptions

*Measuring light (ML) and the estimation of the* ${\bf{\it{\Phi F}}}$. A central feature of PAM ChlF is its capacity to specifically capture the yield of ChlF ($\Phi F$), even under varying ambient illumination (but see below). In other words, PAM-F values are only indirectly influenced by changes in ambient light, through the effects of PQ and NPQ dynamics on $\Phi F$. The specific detection of $\Phi F$ is achieved by exciting fluorescence with µs pulses of constant amplitude (measuring light, ML), followed by subtracting the signal just before the pulse from the signal during the pulse. Accordingly, since the recorded PAM-F value (typically registered in voltage units) originates from the constant ML, its variations can be assumed to be proportional to the quantum yield of ChlF ($\Phi F$) (**Assumption 1**) and can thus be used to calculate PQ and NPQ yields and quenching parameters (Lazár 2015). Critically, for Assumption 1 to hold, it is not sufficient for the ML to remain constant; all factors that affect the rate of energy capture by the population of photosystem II (PSII) units under examination must also be stable. These factors include the geometry and distance between the instrument and the leaf sample, the light absorption properties of the sample (including pigment composition, chloroplast arrangement and thylakoid ultrastructure), and the distribution of excitation energy between the PSII and the less fluorescent photosystems I (PSI) units. Moreover, the contribution of PSI in the recorded PAM-F levels should also be considered before associating PAM-F values to the $\Phi F$ of PSII. Unfortunately, in the absence of data, we did not here attempt to correct our observations for these factors but acknowledge and discuss their potential influence in the text. The user should be aware that variations in these factors can affect the validity of Assumption 1 and, by extension, bias the estimation of ChlF parameters.

*Saturating pulses (SP) and PAM-F levels.* Another key feature of PAM ChlF systems is the use of short (300–800 ms) saturating light pulses. Typically, SP intensity ranges from 6000 to 10,000 μmol m^−2^ s^−1^ PAR, although lower intensities may be sufficient for dark acclimated leaves with low levels of NPQ (Karageorgou et al. 2007). The purpose of the SP is to momentarily reduce all primary electron acceptors of PSII, so that PQ can be assumed to approach zero (**Assumption 2**). This results in a transient increase in the fluorescence yield, registered in PAM-F terms as a rise from the prevailing fluorescence level ($F{^{\prime}}$) to the maximal fluorescence level in the light ($F_M^{\prime}$). Under dark-acclimated conditions, PAM-F values $F{^{\prime}}$ and $F_M^{\prime}$ are instead denoted as minimal (${F_0}$) or maximal fluorescence (${F_M}$), respectively. At night, it can be assumed that all reversible forms of NPQ have relaxed, causing $F_M^{\prime}$ to increase to the higher ${F_M}$ level. Concurrently, all primary electron acceptors in PSII can be assumed to be re-oxidized during nighttime, leading to the full opening of reaction centers and decrease in $F{^{\prime}}$ to the minimal ${F_0}$ level (Maxwell and Johnson 2000; Murchie and Lawson 2013). The significance of the SP method lies in its ability to capture PAM-F both in the presence ($F{^{\prime}}$ and ${F_0}$) and in the absence ($F_M^{\prime}$ and ${F_M}$) of photochemistry, thereby allowing the separation and quantification of PQ and NPQ dynamics with each SP event (Porcar-Castell 2011). Accordingly, diurnal changes in $F{^{\prime}}$ and $F_M^{\prime}$ can be used to calculate and study the reversible dynamics of PQ and NPQ processes in response to diel variations in temperature, radiation and leaf physiological status. To do so, daytime PAM-F values can be compared to the preceding night ${F_0}$ and ${F_M}$. Similarly, day-to-day changes in nighttime ${F_0}$ and ${F_M}$ across the seasons can be used to assess the seasonal dynamics of PQ and NPQ. These dynamics can be associated to photoinhibition of reaction centers and the accumulation of sustained forms of NPQ, in response to environment-driven photosynthetic phenology or plant stress (Ensminger et al. 2004; Porcar-Castell 2011; Verhoeven 2014). To estimate seasonal changes in PQ and NPQ, nighttime ${F_0}$ and ${F_M}$ values are compared to the reference levels ${F_{0R}}$ and ${F_{MR}}$, which represent the ${F_0}$ and ${F_M}$ values in the absence of sustained NPQ and photoinhibition or photodamage of reaction centers (Porcar-Castell 2011). Importantly, using ${F_{0R}}$ or ${F_{MR}}$ to estimate seasonal variations in PQ and NPQ entails that the factors underlying Assumption 1 remain constant throughout the period for which these reference levels are applied, which may extend across several months and require a detailed assessment. For example, periods of leaf development or senescence should be carefully analyzed, as day-to-day changes in ${F_0}$ and ${F_M}$ during these phases are likely to reflect changes in foliar pigment composition and light absorption properties rather than changes in $\Phi F$, and by extension PQ or NPQ.

### General considerations for long-term PAM measurements

Monitoring PAM instrumentation typically consists of multiple fluorometer heads connected to a central control unit, which is responsible for triggering measurements, storing data, and providing remote access. For example, the MONI-PAM used in this study can support up to 16 independent fluorometers, while the Plant Stress Probe by Opti-Sciences (PSP-32, Opti-Sciences, NH, US) can operate a total of 32 fluorometers. These monitoring systems also offer various power supply and communication options for deployment in remote locations. Users should also consider the maximum allowable distance between sensors and control unit, as this sets the maximum radius for plant replication purposes. In contrast, the Monitoring Pen (Monitoring Pen, PSI, Czech Republic) features a compact, standalone design without a central control unit. The following sections are based on our experience with the MONI-PAM system from Walz, but the measuring principles, setup recommendations, considerations for data interpretation, and the LongTermPAM R-package presented here should be broadly applicable and adaptable to users of other long-term monitoring systems.

*ML Settings: Frequency and Intensity.* The frequency of the ML determines the rate at which the instrument collects data (e.g., in the MONI-PAM: 5–25 Hz and up to 100 Hz during SPs), while the intensity of the ML influences the level of recorded PAM-F. Typically, higher ML frequency and intensity improve the signal-to-noise ratio. However, increasing these settings also increases the amount of PAR provided by the ML which can interfere with the estimation ${F_0}$ and should be avoided. Additionally, if the ML intensity is too high, the resulting signal may exceed the dynamic range of the detector during an SP (especially since PAM-F can increase several-fold under these pulses). For example, if the maximum detector range is 4000 mV, then the ML should be adjusted so that ${F_0}$ remains below 680 mV. This threshold value is derived from the relationship ${F_0}$ = (1–0.83) ${F_M}$, assuming a reference maximum quantum efficiency of PSII or ${F_V}$/${F_M}$ = 0.83 after Björkman and Demmig (1987).

Critically, the selected ML settings should remain consistent and valid throughout the entire monitoring period so that PAM-F values acquired at different times can be combined to estimate *PQ* and *NPQ* parameters (See Sect. “On the derivation of parameters from long-term PAM ChlF”). Therefore, if measurements are initialized when leaves are partly downregulated (e.g., during cold periods), users should be aware that $F{^{\prime}}$, and specially $F_M^{\prime}$ values, may increase later in the season potentially leading to signal saturation. Conversely, PAM-F values can decrease significantly from summer to winter in evergreen foliage. In such cases, selecting an ML intensity during summertime that is too low may result in the signal being lost in the noise during wintertime. As a general rule, it is recommended to start measurements during the favourable season when ${F_V}$/${F_M}$ values are around or above 0.8, and targeting for PAM-F values between 300 and 500 mV in the case of the MONI-PAM, or around 10% of the total dynamic range of the instrument. This should ensure a robust signal throughout the seasonal monitoring period, minimizing the risk of either saturation or insufficient signal quality.

*SP Settings: intensity and duration.* The intensity and duration of the SP must be sufficient to meet Assumption 2 and attain the $F_M^{\prime}$ level (or ${F_M}$). At the same time, it is important to minimize the total PAR dose provided by the SPs to avoid long-term photodamage. The SP settings can be optimized by examining the response of $F_M^{\prime}$ to pulses of increasing duration and intensity in leaves acclimated to moderate-to-high light (e.g. Karageorgou et al. 2007). The SP settings beyond which $F_M^{\prime}$ no longer increases indicate the minimum requirements for effective saturation. However, it is recommended to use SP settings that are slightly more intense and longer than this minimum threshold to ensure reliable saturation across a broader range of environmental and physiological conditions. Note that the long-term PAR dosage of the SPs can be also decreased by increasing the interval between measurements (i.e. time between SPs), without compromising the accurate estimation of $F_M^{\prime}$.

*Measurement interval.* The typical configuration for long-term PAM measurements involves point recordings of PAR, ambient or leaf temperature, and PAM-F parameters $F{^{\prime}}$ and $F_M^{\prime}$ using an SP conducted at a pre-set interval, from minutes to hours, with the ML turned off between measurements. Measurement frequency can either be fixed or pre-programmed to vary over time or between measuring heads. In addition, PAM monitoring systems often include actinic illumination options that allow for automated execution of light induction or response curves. While such routines can be useful under specific experimental contexts, we do not recommend them for long-term applications, as they can substantially increase the overall light dosage delivered to the leaf. In general, reducing the interval between measurements (and SPs) results in more data points but also in higher cumulative light exposure, which may induce physiological responses and interfere with the non-invasive nature of monitoring PAM measurements. In this study we used a fixed 30-minute interval, resulting in 48 observations per day. At this frequency, the daily PAR dose from the SP was 307.2 mmol m^−2^ ( = 48 points ×0.8 seconds ×8000 µmol m^−2^ s^−1^), which represents a negligible addition of PAR relative to a sunny summer day, but could become relatively more relevant during winter when daily PAR levels are much smaller. This underscores the importance of minimizing the additional supply of PAR during measurements, particularly in foliage under low light environments. This consideration is relevant given that the rate of photodamage (or photoinhibition) of PSII reactions centres is known to depend on the incoming radiation levels (Tyystjärvi 2008). To mitigate these effects, monitoring PAM systems typically offer programmable options that allow users to reduce overall PAR dosage of measurements, for example, by decreasing the interval between SPs during nighttime or under low-light conditions.

*Impact of temperature on ML intensity*. Current monitoring PAM instrumentation relies on LED technology to provide both ML and SPs, often with configurable LED colour. A critical consideration is that LED light output is temperature dependent, generally decreasing as the LED heats up. This temperature response also varies with LED colour, as blue LEDs tend to be more stable than red or amber LEDs. For example, based on the manufacturer’s technical datasheet (DS65, Luxeon® Revel, Philips), the light output of the blue LEDs used in the MONI-PAM system increases by approximately 2% when moving from 20 °C to −20 °C. In contrast, the light output of red or orange LEDs can vary as much as 25–30% over the same temperature range. Importantly, temperature dependence may also exist in other components of the measuring system which should be characterized or verified with the manufacturer. While small changes in light output typically do not affect the saturating function of the SP, even minor fluctuations in ML output can significantly affect recorded PAMF levels. This may require a correction for field applications where the fluorometers are exposed to substantial temperature variations over the seasons or even within the course of a single day. Generally, ML fluctuations of less than 1% are considered acceptable and do not typically require correction. Moreover, the impact of temperature-dependent ML variation is not uniform across all fluorescence parameters. For example, the estimation of the quantum yield of photochemistry ($\Phi P$) is minimally affected because both $F{^{\prime}}$ an $F_M^{\prime}$ are recorded at the same time and temperature -though the temperature dependence of $F{^{\prime}}$ and $F{^{\prime}}_M$ can be slightly different (see Supplement 1). In contrast, parameters that involve comparing fluorescence measurements taken at different times and temperatures are more susceptible to error. For instance, calculating reversible NPQ at noon (*NPQ*_*r*_= ${F_M}$/$F_M^{\prime}$-1) by combining nighttime ${F_M}$ recorded at 5 °C, with noontime $F_M^{\prime}$ recorded at 20 °C may lead to overestimation of NPQ, as reduced ML output at higher temperature would decrease $F_M^{\prime}$. Similarly, total NPQ (as *NPQ*_*T*_= ${F_{MR}}$/$F_M^{\prime}$-1) measured during cold winter days may be underestimated as it combines a nighttime *F*_MR_ registered during warm summertime nights, with a low-temperature daytime $F_M^{\prime}$ (See Table 1). To address these issues, users can test the temperature dependence and derive correction functions for $F{^{\prime}}$ and $F_M^{\prime}$ using a climate chamber (Supplement 1). Ultimately, the need for such a correction will depend on the temperature sensitivity of the system, the expected temperature range during the monitoring period, and the specific ChlF parameters being evaluated.

**Table 1:** Chlorophyll fluorescence parameters retrieved from MONI-PAM data after Porcar-Castell (2011). Note that the sum of $\Phi NP{Q_s}$ and $\Phi NP{Q_r}$ are here equal to $\Phi NP{Q_T}$ only during nighttime when $F{^{\prime}}$ is equivalent to ${F_0}$, but $\Phi NP{Q_s}$ can be also calculated in daytime by using $F{^{\prime}}$.

Diurnal quenching parameters		Diurnal yield parameters		Seasonal quenching parameters		Seasonal yield parameters	
Total Photochemical quenching	$P{Q_T} = \left( {{{{F_{MR}}} \over {F^{\prime}}} - {{{F_{MR}}} \over {F_M^{\prime}}}} \right)$	Quantum yield of photochemistry	$\Phi P = 1 - {{F^{\prime}} \over { F_M^{\prime}}}$	Sustained photochemical quenching	$P{Q_s} = \left( {{{{F_{MR}}} \over {{F_0}}} - {{{F_{MR}}} \over {{F_M}}}} \right)$	Maximum photochemical efficiency of PSII	$\Phi {P_{max}} = {{{F_V}} \over {{F_M}}} = 1 - {{{F_0}} \over {{F_M}}}$
Total photochemical quenching (fraction)	$q{L_T} = {{\left( {{1 \over {F^{\prime}}} - {1 \over { F_M^{\prime}}}} \right)} \over {\left( {{1 \over {{F_{0R}}}} - {1 \over {{F_{MR}}}}} \right)}}$	Quantum yield of total ${\rm{NPQ}}$	$\Phi NP{Q_T} = {{F^{\prime}} \over {F_M^{\prime}}} - {{F^{\prime}} \over {{F_{MR}}}}$	Sustained photochemical quenching (fraction)	$q{L_s} = {{\left( {{1 \over {{F_0}}} - {1 \over {{F_M}}}} \right)} \over {\left( {{1 \over {{F_{0R}}}} - {1 \over {{F_{MR}}}}} \right)}}$	Quantum yield of sustained NPQ	$\Phi NP{Q_s} = {{{F_0}} \over {{F_M}}} - {{{F_0}} \over {{F_{MR}}}}$
Total Non-photochemical quenching	$NP{Q_T} = \left( {{{{F_{MR}}} \over {F_M^{\prime}}} - 1} \right)$	Quantum yield of reversible NPQ	$\Phi NP{Q_r} = {{F^{\prime}} \over {F_M^{\prime}}} - {{F^{\prime}} \over { {F_M}}}$	Sustained non-photochemical quenching	$NP{Q_s} = \left( {{{{F_{MR}}} \over {{F_M}}} - 1} \right)$	Quantum yield fluorescence and constitutive thermal energy dissipation (seasonal component)	$\Phi F + D = {{{F_0}} \over {{F_{MR}}}}$
Reversible non-photochemical quenching	$NP{Q_r} = \left( {{{{F_{MR}}} \over {F_M^{\prime}}} - {{{F_{MR}}} \over {{F_M}}}} \right)$	Quantum yield of fluorescence and constitutive thermal energy dissipation	$\Phi F + D = {{F^{\prime}} \over {{F_{MR}}}}$				

*Calibration and maintenance.* As with any scientific instrument, regular calibrations and maintenance are essential – ideally performed before each measuring campaign, or at a minimum, once per year. Maintenance procedures should include visual inspection of the fluorometer head and leaf clips, and checks on all optical components, cables and connections for signs of damage or wear. For detailed guidance on adjusting the ML sensitivity and calibrating the PAR and temperature sensors in the MONI-PAM system, users are referred to Supplements 2 and 3. Getting familiar with the instrument manuals and contacting the manufacturer’s technical support when in doubt is usually the first step towards successful measurements.

**Fig. 1:**
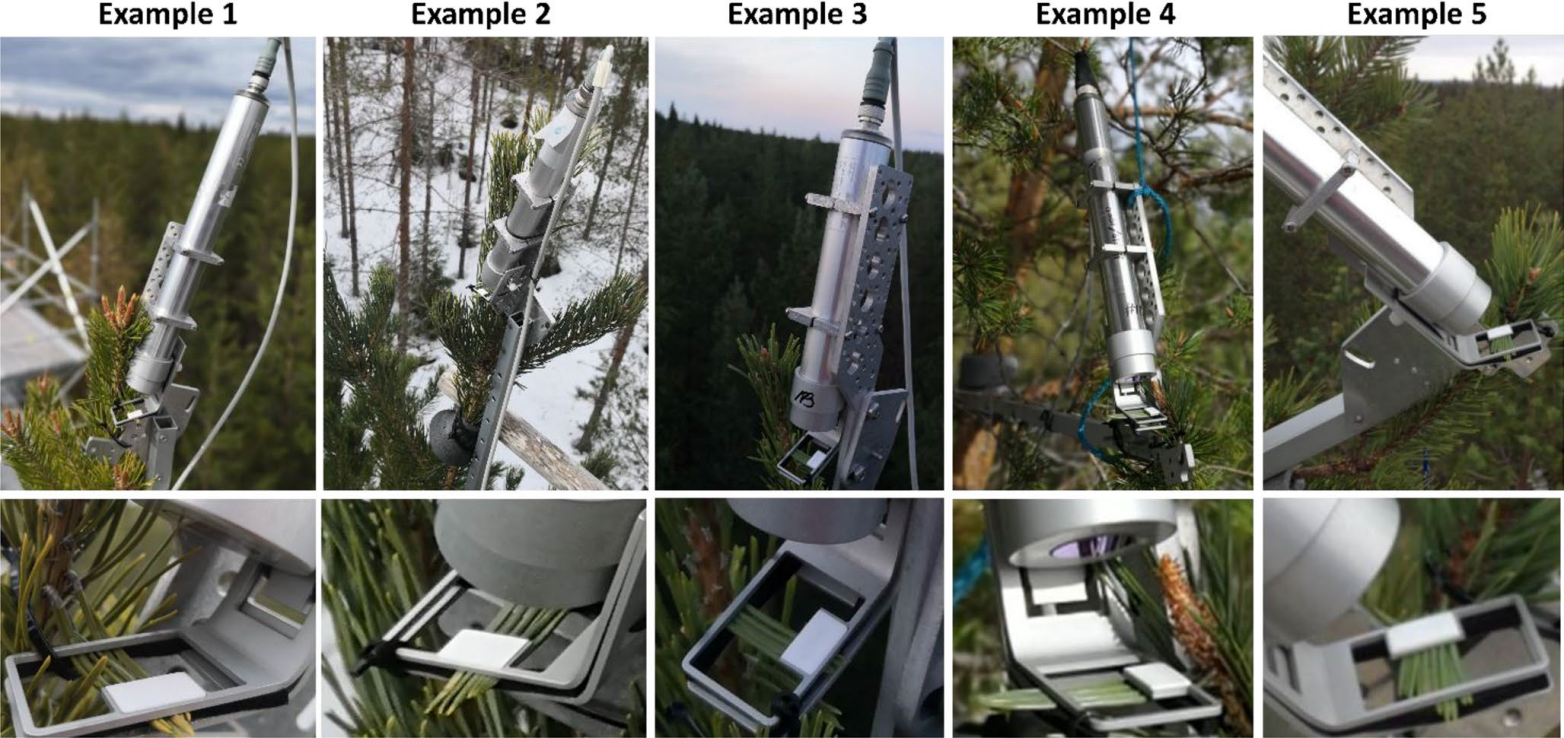
Examples of MONI-PAM fluorometer installations in pine trees. the images illustrate the flexibility of the custom field-mounting supports, enabling secure installation in various configurations. Examples 1, 2 and 3 show installations on vertically oriented top shoots, while examples 4 and 5 depict setups on more horizontally oriented lateral shoots

*Field Installation and mounting supports.* While monitoring PAM fluorometers can be mounted on simple tripods for short term or indoor applications, long-term field installations require specialized attachments and supports to ensure that the relative geometry between the measuring head, leaf sample, and branch remains stable – even under windy conditions. To this end, specialized supports were designed for mounting MONI-PAM fluorometers (Fig. 1, S1) in woody vegetation. Notably, the design could be likely adapted to other cylindrical-type fluorometers, such as the Plant Stress Probe from Opti-Sciences. The design of the supports was developed to provide flexibility, allowing users to mount fluorometers in a variety of configurations to accommodate different shoot architectures (see Fig. 1). A detailed description of the support components, including links to the STEP/CAD files for manufacturing and practical instructions for field installation, is provided in Supplement 4 and supplementary Fig. S1.

## On the derivation of parameters from long-term PAM ChlF

Processing long-term PAM ChlF data involves calculating parameters that require the combination of PAM-F levels (e.g. $F{^{\prime}}$, ${F_0}$, ${F_M}$, ${F_{MR}}$) acquired at different points in time. A distinctive feature of long-term PAM ChlF datasets is that they span across multiple temporal scales. This allows researchers to investigate both short-term or diurnal regulation of the light reactions, as well as long-term or seasonal regulation in response to plant stress or photosynthetic phenology. This means that derived parameters should remain internally consistent to ensure meaningful comparisons over time, facilitate quantitative analysis, and support integration into mathematical models. To address this need, Porcar-Castell (2011) compiled, updated and derived a suite of ChlF parameters to facilitate the quantitative analysis of long-term PAM datasets. These parameters are used in this communication to demonstrate the potential of long-term PAM measurements and briefly introduced below and in Table 1.

Non-photochemical quenching has been widely estimated based on the Stern-Volmer *NPQ* parameter (Bilger and Björkman 1990). The *NPQ* parameter provides a measure of the rate constant of regulated thermal energy dissipation in the antenna (*k*_*NPQ*_, in s^−1^) relative to the sum of the first order rate constants of fluorescence and constitutive thermal energy dissipation (*k*_*F*_ + *k*_*D*_, respectively) (Kramer et al. 2004; Porcar-Castell 2011). As such, the *NPQ* parameter represents a meaningful and quantitative metric to study and model the light reactions of photosynthesis. This is in contrast to the earlier defined *qN* parameter (Bilger and Schreiber 1986; Krause and Weis 1991; Roháček 2002), which reflects the quenching of variable fluorescence rather than the quenching of maximum fluorescence, as *NPQ* does. Porcar-Castell (2011) adapted the calculation of *NPQ* to consider its diurnal and seasonal components, using the recorded nighttime ${F_M}$ and a summer reference ${F_{MR}}$, respectively, following the conceptual approach introduced by Maxwell and Johnson (2000). In turn, to address PQ dynamics, Porcar-Castell (2011) introduced the $q{L_T}$ parameter which describes the total fraction of open and functional reaction centers of Photosystem II. This parameter is conceptually analogous to the $qL$ parameter introduced by Kramer et al. (2004) but distinguishes two kinetic components of photochemical quenching. Specifically, $q{L_T}$ is the product of $q{L_r}$, the fast and reversible component of photochemical quenching that denotes the fraction of open reaction centers, and $q{L_s}$, the slow and sustained component indicating the fraction of functional (i.e. non-photodamaged or non-photoinhibited) reaction centers.

A challenge in analyzing PQ and NPQ dynamics is that $$q{L_T}$$ (a fraction) and *NPQ* (a relative rate constant) differ in both units and interpretation. To address this, Porcar-Castell (2011) proposed the *PQ* parameter – formulated as a relative rate constant, analogous to *NPQ*. *PQ* is calculated as the product of $$q{L_T}$$ and the maximum photochemical quenching (*PQ*_max_), and like *NPQ* it shares the denominator (*k*_*F*_+*k*_*D*_). As $$q{L_T}$$ declines, *PQ* correspondingly decreases. In turn, *PQ*_max_ is defined as *k*_*PSII*_/(*k*_*F*_+*k*_*D*_) and can be derived from the reference maximum photochemical yield, $$({F_V}$$/$${F_M}{)_R}$$, as *PQ*_max_ = $$({F_V}$$/$${F_M}{)_R}$$/(1-$$({F_V}$$/$${F_M}{)_R}$$) =$$ {F_{MR}}$$/$${F_{0R }}$$-1. Thus, for the broadly used reference $${F_V}$$/$${F_M}$$ = 0.83 (Björkman and Demmig 1987), *PQ*_max_ would equate 4.88. This formulation enables direct comparison between dynamics of PQ and NPQ within a unified quantitative framework, whereas $$qL$$ provides insights into the functionality and openness of PSII reaction centers.

Finally, PAM-F data can be used to assess quantum yields of photochemistry ($\Phi P$), reversible, sustained and total thermal dissipation (*ΦNPQ*), as well as the combined yield of chlorophyll fluorescence emission and constitutive thermal dissipation ($\Phi F + D$), also referred to as the yield of other energy losses ($\Phi NO$) (Kramer et al. 2004). In the current communication and the accompanying R package we focus on the parameters summarized above and listed in Table 1. However, users may wish to explore additional parameters depending on their specific goals.

## Data processing with R package “LongTermPAM”

**Fig. 2:**
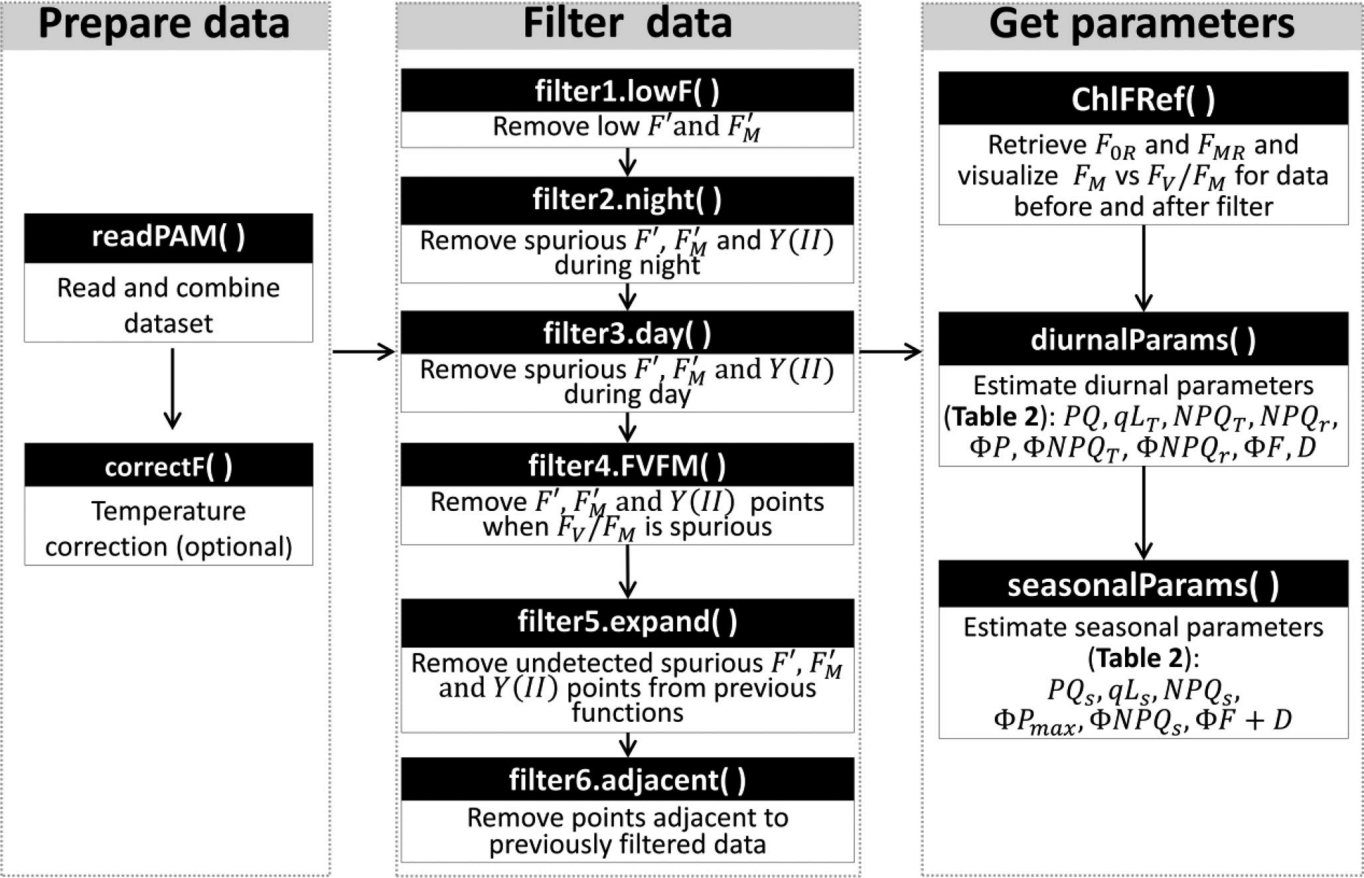
The LongTermPAM R package workflow and its three main phases. Black headers indicate the relevant package functions along with a brief description. (1) prepare data: the package can read CSV or txt files recorded by MONI-PAM system using the function readPAM(). The $F{^{\prime}}$ and $F_M^{\prime}$ can then be temperature corrected using a correction function: correctF(). (2) filter data: functions starting with ‘filter’ are used to identify and remove spurious values of $F{^{\prime}}$, $F_M^{\prime}$, and $Y\left( {II} \right)$. (3) get parameters: ChlF parameters such as *NPQ* and *PQ* can be obtained by the diurnalParams() or seasonalParams() function

Field measurements are inherently exposed to environmental variability, which is particularly relevant when using optical methods such as PAM fluorometry. Meteorological factors including rain, snow, dew, ice formation, or wind; as well as biotic factors like insects obstructing the optical path, can interfere with measurements. These interferences can affect the transmission of ML and SP from the sensor to the sample, or the transmission of the ChlF from the sample to the sensor. It is therefore essential to detect and exclude these spurious observations. While manual identification and removal of such outliers is possible, it can be very time consuming if datasets cover long periods of time or span across many measuring heads. Moreover, manual filtering introduces subjectivity, especially when performed by different users. To address these challenges, we developed LongTermPAM R-package, which provides an automated approach for filtering and processing long-term PAM ChlF datasets. The workflow includes three steps (Fig. 2): (1) reading and preparing the data (Prepare data), (2) automatically identifying and removing spurious or unreliable data points (Filter data), and (3) calculating ChlF parameters based on the filtered dataset (Get parameters). In addition, the package includes several data visualization tools to help the users assess the filtering process and fine-tune the filtering criteria. A brief overview of the package can be found in Supplement 5 and the full documentation is available at the GitHub repository (https://github.com/chaoxzhang/LongTermPAM). Users can also explore example outputs and visualizations in zenodo (https://zenodo.org/records/14961722). Although not tested, the package should be easily adaptable to other instrument data formats. Note also that the R package uses $Y\left( {II} \right)$ to represent the quantum yield of photochemistry instead of $\Phi P$, to maintain consistency with the MONI-PAM data output format.

To introduce the logic of the data filtering process and to provide a reference for the following chapters, it is useful to define three core PAM-F parameters involved in the filtering routine: PAM $F{^{\prime}}$ Eq. (1), $F_M^{\prime}$ Eq. (2), and $\Phi P$ Eq. (3), as follows:

1 $$\eqalign{& {F^\prime } = PA{R_{ML}} A{\alpha _{II}}\beta {{{k_F}} \over {{k_F} + {k_D} + {k_{NPQs}} + {k_{NPQr}} + q{L_s}q{L_r}{k_{PSII}}}}+ {C_{PSI}} \cr} $$

2 $$F_M^{\prime} = PA{R_{ML}} A {\alpha _{II}} \beta {{{k_F}} \over { {k_F} + {k_D} + {k_{NPQs}} + {k_{NPQr}}}}+{C_{PSI}}$$

3 $$\Phi P = {{F_M^{\prime} - F{^{\prime}}} \over {F_M^{\prime} - {C_{PSI}}}} = {{q{L_s} q{L_r} {k_{PSII}}} \over {{k_F} + {k_D} + {k_{NPQs}} + {k_{NPQr}} + q{L_s} q{L_r} {k_{PSII}}}}$$

where *PAR*_*ML*_ is the amount of ML PAR reaching the leaf surface (in µmol m^−2^ s^−1^), *A* is the PAR absorption by photosynthetic pigments in the leaf at the wavelength of the ML, *α*_*II*_ is the fraction of absorbed radiation directed to PSII units at the wavelength of the ML; $$\beta $$ is a parameter that clumps together the probability of fluorescence photons escaping the leaf and reaching the sensor, the area under examination, and the sensor sensitivity [mV m^−2^ µmol^−1^ s^1^], and *C*_*PSI*_ is the contribution of PSI in the PAM-F signal, typically assumed to remain unaffected by the SP (but see Schreiber and Klughammer (2021)). Furthermore, the quantum yields of fluorescence Eqs. (1 and 2) and photochemistry Eq. (3) are expressed as the quotient of the respective first order rate constants [s^−1^], where *k*_*F*_, *k*_*D*_, *k*_*NPQr*_, *k*_*NPQs*_, and *k*_*PSII*_ denote the rate constants for fluorescence, constitutive thermal dissipation, reversible and sustained non-photochemical quenching or regulated thermal dissipation (NPQ), and the intrinsic rate of photochemistry in functional and open PSII reaction centers (*k*_*PSII*_), respectively. While the $$q{L_s}$$and $$q{L_r}$$ parameters denote the sustained and reversible components of photochemical quenching as described above (Porcar-Castell 2011). Clearly, disturbances like dew, snow or wind-induced movement of leaves out of the frame will tend to proportionally decrease $$F{^{\prime}}$$ and $$F_M^{\prime}$$ in a similar fashion, as they affect the level of *PAR*_*ML*_ or $$\beta $$ – either by lowering the amount of ML reaching the leaf surface or decreasing the area of sample visible to the sensor. In contrast, these disturbances should not affect the estimation of the quantum yield of photochemistry ($$\Phi P$$) Eq. (3), as they cancel out. However, in practice, disturbances can also impact $$\Phi P$$, due to decreasing signal-to-noise ratios if absolute levels of PAM $$F{^{\prime}}$$ and $$F_M^{\prime}$$ are very low. Likewise, it should be noted that the effect of the PSI contribution (*C*_*PSI*_) to the measured PAM-F levels does not cancel out when estimating $$\Phi P$$ Eq. (3). Overall, the differential response of $$F{^{\prime}}$$, $$F_M^{\prime}$$ and $$\Phi P$$ to external disturbances is one of the criteria used by the filtering process to identify abnormal observations. Another important criterion used by the filtering process is the rate of change. For example, while it is possible to register rapid changes in $$F_M^{\prime}$$ during daytime in response to fluctuations in illumination, rapid changes in $$F_M^{\prime}$$ (or $$ {F_M}$$) during nighttime typically indicate rainfall, dew formation or snowfall. Gradual day-to-day changes in nighttime $${F_M}$$ can suggest accumulation or relaxation of NPQ_s_, especially if accompanied by changes in nighttime $${F_V}$$/$${F_M}$$. Alternatively, if $${F_V}$$/$${F_M}$$ remains stable, these changes may point to a leaf sample that is gradually moving out the sensor frame. Logical filters are implemented to flag these abnormal observations, and to un-flag them once levels get back into the expected range.

## Description of field datasets

Two case-study datasets were collected at the SMEAR II (Station for Measuring Ecosystem-Atmosphere Relations) in Hyytiälä, southern Finland (61°51′N, 24°17′E, 181 m above sea level) and used here to discuss the applicability of long-term measurements and demonstrate the performance of the LongTermPAM R-package. Fluorometers were installed in fully developed and current-year needles of Scots pine (*Pinus sylvestris* L.) and Norway spruce (*Picea abies* L.) from top and low canopy sections, with the help of scaffolding towers. A first dataset was collected from 24.8.2014 to 1.9.2015 and included data from five fluorometers mounted in three different pine trees: three in the top canopy (first 3 whorls) of each tree (Data codes: Pine5, Pine6TOP, and Pine7TOP), and two in the lower canopy (Pine6LOW and Pine7LOW). A second dataset was collected from 3.11.2016 to 4.9.2017 and included data from seven fluorometers mounted in three pines and three spruce trees: three in top canopy pines (Pine1, Pine2, and Pine3), two in needles from two short understory spruce trees (Spruce1, Spruce3), and two in the top (Spruce2TOP) and low canopy of a spruce tree (Spruce2LOW). This second dataset was collected using two separate MONI-PAM systems, each with its own control unit (MONI-DA, Heinz Walz GmbH, Germany). For simplicity, measurements were here collected using the internal clock function of the MONI-DA which automatically triggered an SP every 30 minutes. At each measuring point, each fluorometer recorded the amount of incoming PAR, ambient temperature, and the prevailing ($$F{^{\prime}}$$) and maximal ($$F_M^{\prime}$$) PAM fluorescence levels, which could later be attributed to $$ {F_0}$$, $${F_M}$$, $${F_{0R}}$$, or $${F_{MR}}$$, as appropriate. It is worthwhile noting that the spruce trees measured in this study were slightly shorter than the surrounding trees so the light environment at the top of the spruce trees was slightly less sunny that that at the top of the pine trees. More details on these two towers and their trees can be found in Rajewicz et al. (2023).

## Results and discussion

**Fig. 3:**
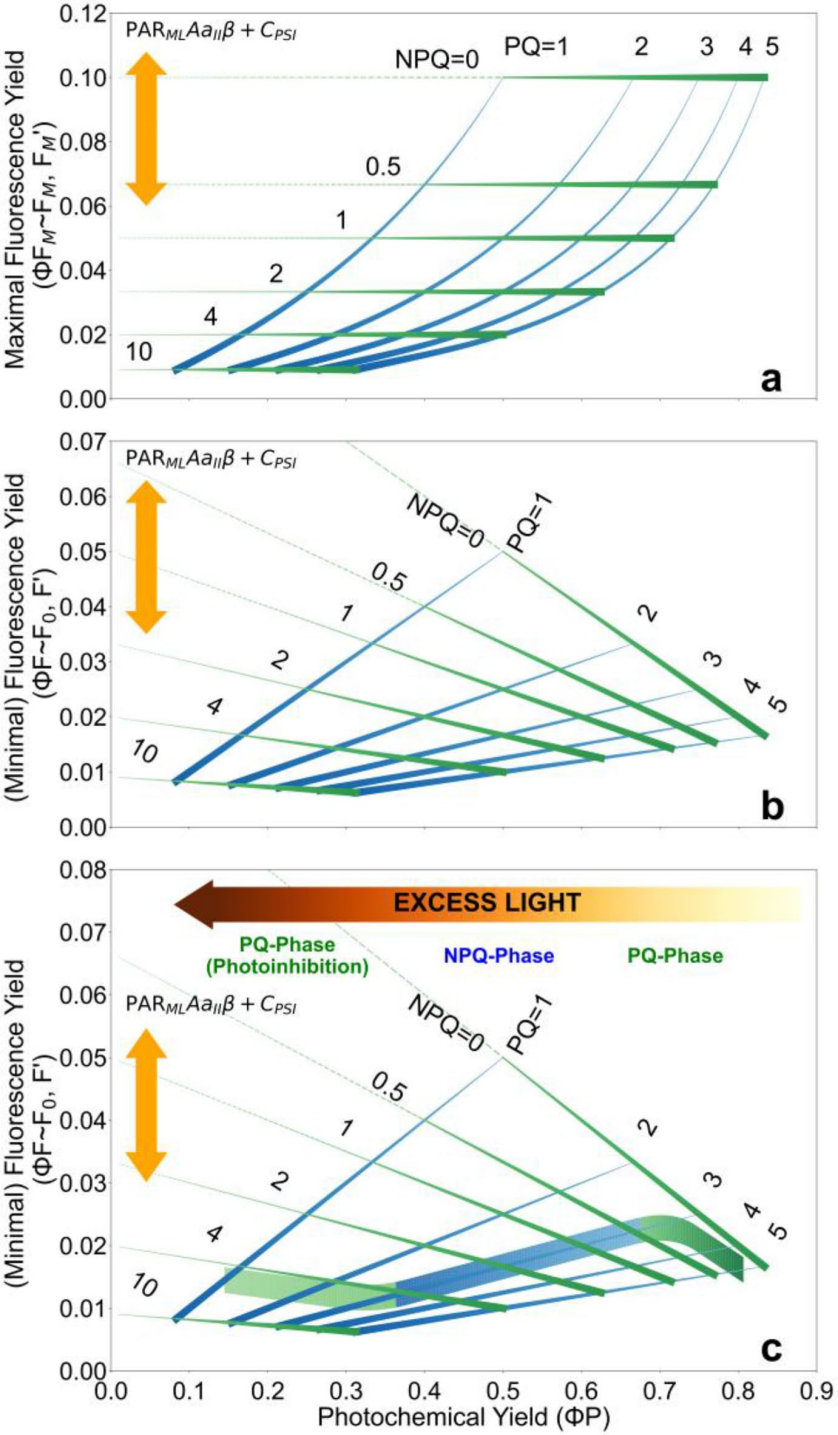
Theoretical relationships between the photochemical yield of PSII (*ΦP*) and PAM parameters $F_M^{\prime}$ or ${F_M}$ (**a**) and $F{^{\prime}}$ or ${F_0}$ (**b**), underlying the three main phases of the relationship between PAM-F and $\Phi P$ (**c**), within a space of PQ and NPQ variation. Lines are calculated after Porcar-Castell et al. (2014) based on a lake model assumption and a theoretical maximal fluorescence efficiency of PSII of 10%, after Barber et al. (1989), whereby $\Phi F$ = 0.1/(1+NPQ+PQ), $\Phi {F_M}$ = 0.1/(1+NPQ), and $\Phi P$ = PQ/(1+PQ+NPQ). Blue lines of increasing thickness represent responses to increasing NPQ estimated at constant levels of PQ. Green lines of increasing thickness represent responses to increasing PQ estimated at constant levels of NPQ. Although the relationship between $\Phi F$ and $\Phi P$ can take multiple forms within the PQ-NPQ space, it often follows a sequential pattern in response to excess light as exemplified in panel (**c**). This includes an initial photochemical phase (PQ-Phase) where the decrease in PQ dominates the relationship; followed by an NPQ-Phase where NPQ gradually increases and dominates the relationship; and eventually a second PQ-Phase where PQ continues to decrease once the capacity of NPQ is either saturated or inhibited. Finally, since *PAR*_*ML*_, *A*, *α*_*II*_, *β* or the contribution of PSI to ChlF (*C*_*PSI*_) can affect PAM-F levels, variation in these factors will increase or decrease PAM-F levels (vertical yellow arrows), and only when they remain constant we can attribute variations in PAM-F to those in $\Phi F$

Before discussing the performance of the LongTermPAM R-package and presenting the results, it is useful to revisit the mechanistic relationship between the PAM $F{^{\prime}}$ and $F_M^{\prime}$ values -associated with the prevailing ($\Phi F$) and maximal ($\Phi {F_M}$) quantum yields of fluorescence-, and the photochemical yield of photosystem II ($\Phi P$), which captures the dynamic regulation of photosynthetic efficiency. Thanks to the compatibility between *NPQ* and *PQ* parameters discussed earlier, it is possible to calculate a theoretical grid of PQ and NPQ variation that illustrates the domain within which the relationship between $\Phi F$ or $\Phi P$ can occur (Porcar-Castell et al. (2014), Fig. 3). This framework is important for understanding how PQ and NPQ processes interact to influence PAM-F signals, how the $\Phi F$- $\Phi P$ relationship evolves over time in response to these dynamics, and how variations in factors such as *PAR*_*ML*_, *A*, *a*_*II*_, *β* or *C*_*PSI*_ may interfere with the relationships (Fig. 3). Ultimately, this understanding can help users to assess the quality of the data and conduct a critical interpretation.

**Fig. 4:**
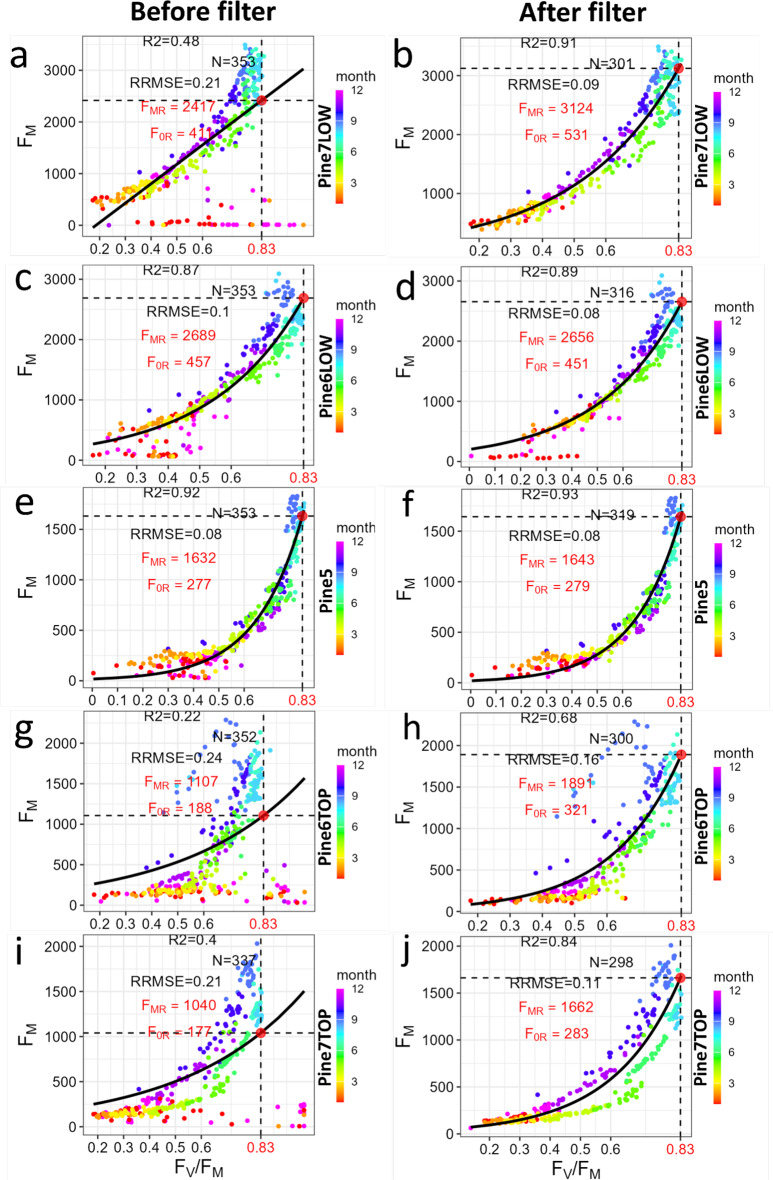
Correlations between *F*_*M*_ and *F*_*V*_/*F*_*M*_ before (left column) and after (right column) data filtering for the 2014-2015 dataset collected in Scots pine needles. Diagnostic statistics are derived from either a linear or non-linear regression model, including R^2^- correlation coefficient, N-number of points used to fit the model, RRMSE - relative root mean square error. The vertical dashed line indicates the selected reference *F*_*V*_/*F*_*M*_ (set here to 0.83) and the horizontal dashed line the corresponding *F*_*V*_/*F*_*MR*_ automatically estimated from the fitted model

**Fig. 5:**
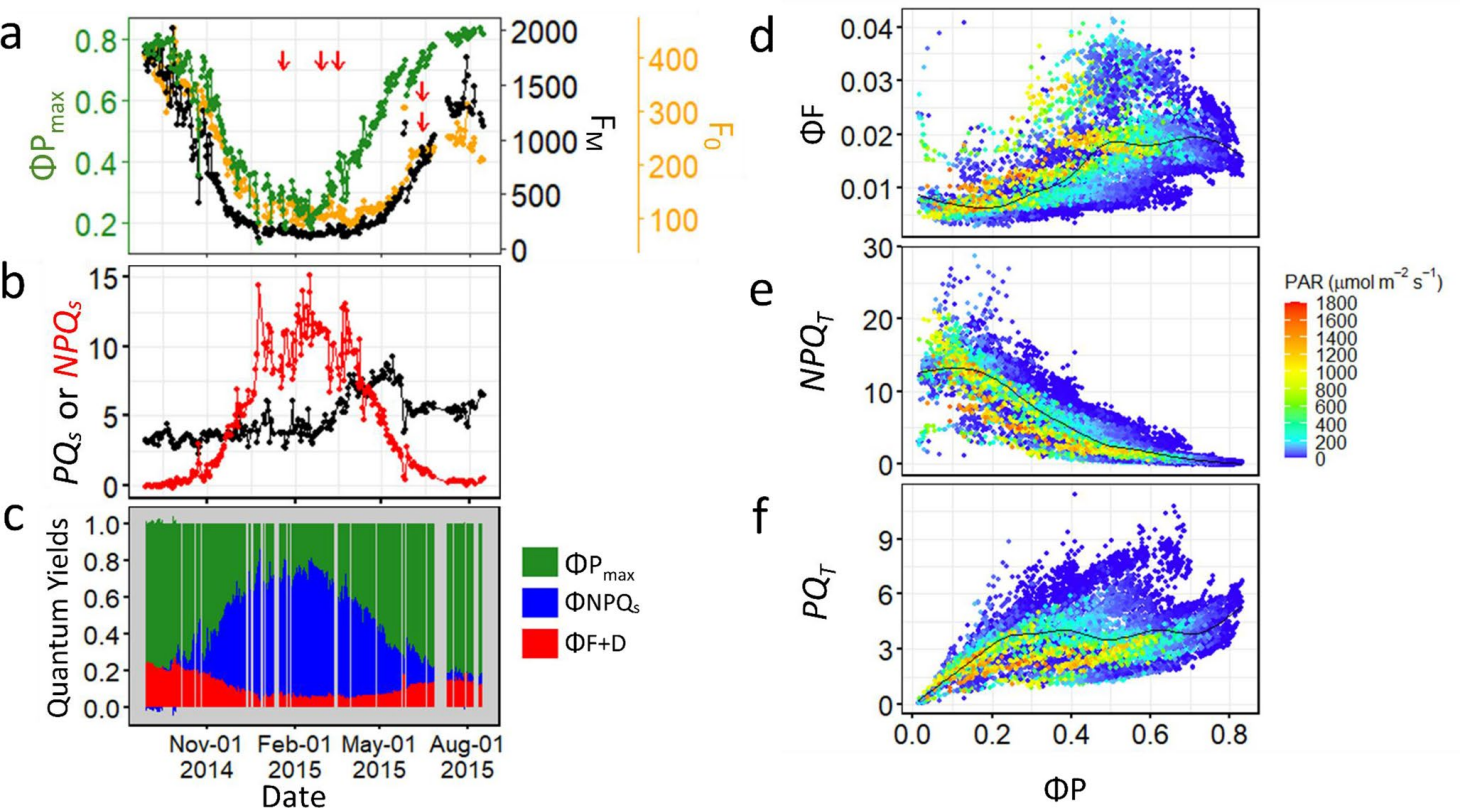
Seasonal variations in fluorescence parameters and the relationship between Φ*P* and Φ*F*,*PQ*_*T*_, *NPQ*_*T*_ in relation to PAR. Seasonal variation in *F*_*0*_, *F*_*M*_ and Φ*P*_*max*_ (or *F*_*V*_/*F*_*M*_) (**a**), *PQ*_*s*_ and *NPQ*_*s*_ (**b**), and Φ*P*_*max*_, Φ*NPQ*_*s*_, and Φ*F+D* (**c**), and relationships between Φ*F* (**d**), *NPQ*_*T*_ (**e**) and *PQ*_*T*_ (**f**) with Φ*P* at different levels of PAR, including all measuring points for the observation period of 2014-2015. In d, Φ*F* is calculated as 0.1*Φ*F+D*, assuming a theoretical maximum fluorescence yield in PSII of 10%. All the data are from tree Pine7TOP. Results from the other measuring heads and observational period can be found in the Supplementary Figs. S4-S7. For panels d-f, we applied generalized additive models (GAM) with integrated smoothness for curve fitting, using the geom_smooth() function from the ggplot2 R package. In panel a, the five red arrows correspond to the dates shown in Fig. 6

Firstly, our theoretical analysis indicates that the relationship between maximal $\Phi {F_M}$ and $\Phi P$ should be non-linear under the influence of NPQ (blue lines in Fig. 3a), whereby both $F_M^{\prime}$ and $\Phi P$ decrease as NPQ levels increase. In turn, since PQ does not influence $\Phi {F_M}$ (**Assumption 2**) but does affects $\Phi P$, the effect of PQ is that of shifting the relationship between $\Phi {F_M}$ and $\Phi P$ right and left along the $\Phi P$-axis (Fig. 3a). In contrast, the effect of PQ and NPQ on the relationship between $\Phi F$ and $\Phi P$ (Fig. 3b) is more complex. While increases in either PQ or NPQ reduce $\Phi F$, they have opposing effects on $\Phi P$. Consequently, the relationship between $\Phi F$ and $\Phi P$ can be positive (when dominated by NPQ variation), negative (when dominated by PQ variation), or neutral (when PQ and NPQ variations offset each other) (see Fig. 3c and sections below). In addition to PQ and NPQ, it is important to note that variations in PAM-F levels can be also affected by *PAR*_*ML*_, *A*, *α*_*II*_, *β* and *C*_*PSI*_ so that an increase or decrease in these parameters will result in a proportional increase or decrease in the measured PAM-F values (yellow arrows in Fig. 3). Therefore, only when these parameters remain stable can changes in PAM-F be confidently attributed to variations in $\Phi F$ (**Assumption 1**).

### Performance and limitations of the data filtering

The LongTermPAM R-package includes a suite of data visualization functions to help users evaluate the performance of the data filtering process and explore the temporal dynamics of ChlF parameters calculated by this R package (Table 1). One key diagnostic plot that can be used for quality control is the relationship between daily maximal $$\Phi P$$ (i.e. $${F_V}/{F_M}$$) and the nighttime $$\Phi {F_M}$$ (via PAM-$${F_M}$$) (Fig. 4, S2). In Scots pine needles, and during the 2014–2015 period (Fig. 4, see Fig S3 for the 2016–2017 dataset), this relationship exhibited the expected non-linear pattern as described in Fig. 3a. However, prior to filtering, the dataset contained numerous outlier observations deviating from the expected pattern (Fig. 4), which reflected the impact of meteorological factors such as snow, ice or dew. As shown in Fig. 3a, the relationship between $$\Phi P$$ and $$\Phi {F_M}$$ can broaden or display hysteresis under variable PQ levels. Additionally, deviations may also arise from changes in the effective sample area (e.g. if part of the leaf moves outside the field of view), in foliar pigment contents and leaf absorption (A), in the energy partitioning between PSII and PSI (*α*_*II*_), or in the contribution of PSI to the PAM-F signal (*C*_*PSI*_). Under such conditions, it may be advisable to estimate a new $${F_{MR}} $$ level that reflects the altered state of *PAR*_*ML –*_ *A*_*leaf* –_ *α*_*II*_ *-β -C*_*PSI*_ – thus preserving the interpretability of PAM-F as a proxy of fluorescence yields. In other words, rather than applying a single $${F_{MR}}$$ level to the entire dataset, as shown in the diagnostic plots of Figs. 4 and S3, users may consider dividing the dataset into two or more subsets, each with its own $${F_{MR}}$$ level. For example, Pine5 (Fig. 4f) appears to follow a consistent $${F_{MR}}$$, whereas data from Pine7TOP (Fig. 4j) might consist of three separate subsets, each potentially requiring its own $${F_{MR}}$$ level. Nevertheless, caution should be taken when partitioning datasets, as introducing too many subsets without a clear rationale (e.g., sample replacement) can obscure the capacity of the measurements to detect dynamics of actual regulatory processes.

In our experimental site, seasonal variation in needle chlorophyll content for Scots pine and Norway is relatively modest, typically ranging from 10 to 15% between winter and summer (Rajewicz et al. 2023; Zhang et al. 2019), with only minor changes observed in total leaf PAR absorption (Zhang et al. 2019). These slight seasonal shifts do not correlate either with PAM derived *PQ*_*s*_ or *NPQ*_*s*_ values for Scots pine needles at the same location (Zhang et al. 2019), supporting the assumption of a constant *A* in those conditions. However, this assumption may not hold for other species or under different conditions. Similarly, variations in *α*_*II*_, in *β*, or the contribution of PSI to the measured PAM-F (*C*_*PSI*_) can also affect the measured $F{^{\prime}}$ and $F_M^{\prime}$ levels, thereby requiring additional investigation and corrections. Without additional observations, it is not possible to determine whether the clusters of datapoints in the diagnostic plots (Fig. 4) were the result of meteorological artifacts (and therefore should be filtered out), shifts in *PAR*_*ML –*_ *A* – *α*_*II*_ *-β-C*_*PSI*_ (which would necessitate adjusting ${F_{MR}}$), or true reflections of physiological changes in PQ and NPQ. Clearly, supplementary data including a time lapse camera, or even better a spectral camera, could assist in evaluating the different scenarios.

Overall, while the automated filtering with LongTermPAM R-package was able to effectively filter a large portion of spurious data, a clear trade-off emerged between the intensity of the filtering (adjustable via parameters in Table S1) and the extent of data loss incurred during the process. Therefore, diagnostic plots generated by the package (Figs. 4 and S3) should be carefully examined and considered when interpreting the ChlF data in terms of PQ and NPQ. Importantly, users should remember also that changes in *PAR*_*ML –*_ *A* – *α*_*II*_ *-β-C*_*PSI*_ can significantly bias the estimation of ChlF parameters (Table 1 and Fig. 3).

### Resolving the diurnal and seasonal variations in PAM ChlF parameters

The seasonal variation in ${F_0}$, ${F_M}$ and ${F_V}$/${F_M}$ (or ΦP_max_) recorded in our case-studies was typical of boreal forest evergreen foliage, characterized by a pronounced decline in ${F_0}$ and ${F_M}$ levels during the winter months, accompanied by reduced nighttime ${F_V}$/${F_M}$ and increased *NPQ*_s_ levels (Fig. 5a,b; see also Figs. 4 and S5 for the rest of observations including spruce). These patterns are consistent with multiple studies at the same site (Grebe et al. 2024; Porcar-Castell 2011; Rajewicz et al. 2023; Zhang et al. 2019), as well as previous research in Northern evergreen forests (Ensminger et al. 2004; Kim et al. 2021; Pierrat et al. 2024). In this study, the broad spatial and temporal coverage provided by multiple PAM fluorometers allowed us to capture the differential regulatory responses within plant canopies. For example, wintertime *NPQ*_s_ levels were consistently higher in the more exposed top canopy relative to the more shaded foliage (Fig. S4), and species-specific differences were also evident (Fig. S5), with spruce needles exhibiting lower levels of downregulation, albeit with a similar spring recovery trend. These species-specific differences can be partly explained by the differences in the light environment, as the measured spruce foliage experienced slightly more shaded conditions (Rajewicz et al. 2023). The light environment has been shown to influence the level of downregulation of the light reactions of photosynthesis in overwintering evergreens. Exposed foliage tends to exhibit higher levels of xanthophyll-cycle pigments and associated de-epoxidation states, greater *NPQ*_S_, and lower ${F_V}$/${F_M}$ during the cold season relative to shaded foliage (Porcar-Castell et al. 2008b; Sveshnikov et al. 2006). Similarly, differences in downregulation patterns between species have also been reported in earlier studies (e.g. Linkosalo et al. 2016; Rajewicz et al. 2023), although not with the temporal resolution and replication enabled by continuous field monitoring with PAM systems.

It is also important to note that the dynamics of sustained PQ (via the *PQ*_S_ parameter), which reflect seasonal changes in the relative rate constant of photochemistry, did not always align with previous observations in the same site. Typically, *PQ*_S_ tends to decrease during the early spring when temperatures are low and radiation levels high (Porcar-Castell 2011; Rajewicz et al. 2023; Zhang et al. 2019). Also, values of *PQ*_S_ exceeded at times the theoretical maximum of 4.88 set by our (${F_V}$/${F_M}$)_R_ level of 0.83 (see Sect. “On the derivation of parameters from long-term PAM ChlF”), which was selected on the basis of recorded summer levels (Figs. 5b and S4f-j) and consistent also with Björkman and Demmig (1987). The reason for the overestimation in *PQ* is partly due to our demonstrative use of a single ${F_{MR}}$ level across the entire measuring period, rather than dividing the dataset into subsets. Additionally, residual spurious observations, such as outlier values that remained after filtering Pine6TOP, may have skewed the estimation of ${F_{MR}}$. These discrepancies highlight the importance of critically assessing the data and the validity of Assumption 1, particularly in the context of PQ estimations. In contrast, ${F_V}$/${F_M}$ (Fig. 5a, c) is substantially less sensitive to departures from Assumption 1 Eq. (3).

Overall, despite of the large variability in environmental conditions and the complex interplay between diurnal and seasonal regulatory processes, a general pattern of variation between $\Phi P$ (including nighttime ${F_V}$/${F_M}$) and the estimated fluorescence yield ($\Phi F$) could be observed (black lines in Figs. 5d, as well as Figs. S6 and S7). Importantly, these patterns are consistent with the three distinct phases illustrated by our theoretical framework presented in Fig. 3c (see also examples in Figs. S3 and S4), supporting the validity of the framework. First, a photochemical phase (PQ-phase) emerged at moderately high $\Phi P$ levels under reasonably low PAR, where $\Phi F$ increases as $\Phi P$ decreases (Fig. 5d). This phase occurs under conditions of relatively constant *NPQ* (Fig. 5e) but decreasing *PQ* (Fig. 5f). Second, a non-photochemical phase (NPQ-Phase) dominated the intermediate range of $\Phi P$ values, during which both $\Phi F$ and $\Phi P$ decrease simultaneously. This phase occurs under conditions of relatively constant *PQ* (Fig. 5f) and increasing *NPQ* (Fig. 5e). Third, a second PQ-phase was observed where $\Phi P$ and $\Phi F$ once again exhibit an inverse relationship. This phase typically occurs under conditions when the NPQ capacity of the leaf is either saturated or inhibited and we address it here as the photoinhibitory phase. Such a pattern has been previously documented under light conditions exceeding those to which the leaves were acclimated (Cendrero-Mateo et al. 2015), or when NPQ is impaired by external factors such as high temperatures (Martini et al. 2022) or, in our dataset, low winter temperatures (Figs. S6u-y and S7ac-ai).

**Fig. 6:**
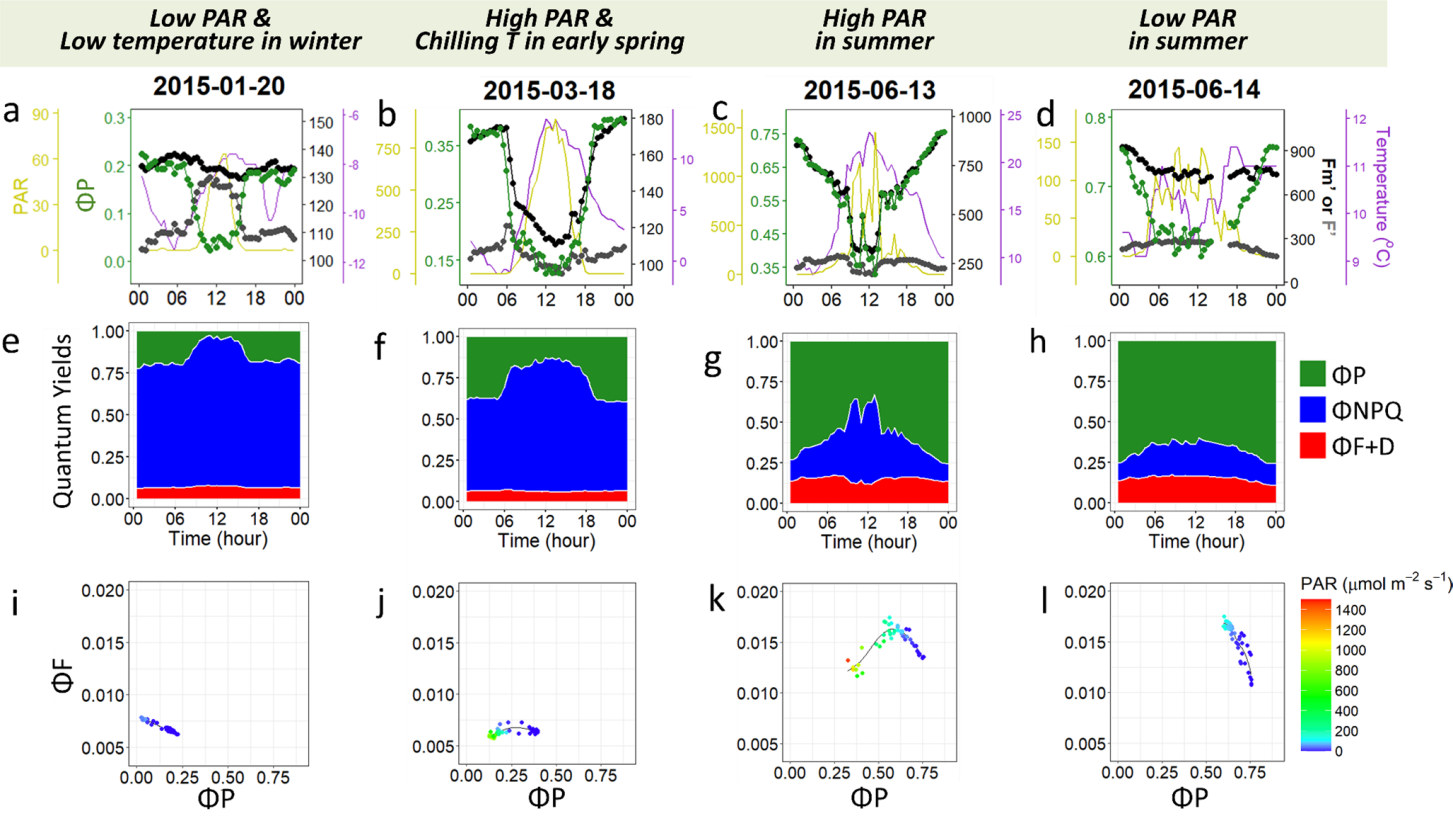
Specific examples of diurnal variations in chlorophyll fluorescence parameters from tree Pine7TOP, 2014-2015. In **i**-**l**, we applied generalized additive models (GAM) with integrated smoothness for curve fitting, using the geom_smooth() function from the ggplot2 R package. $\Phi F$ is calculated as 0.1*$\Phi F + D$, assuming a theoretical maximum fluorescence yield in PSII of 10%

It is important to note that these phases do not necessarily need to occur in the same sequence and will depend on the prevailing physiological and environmental conditions. For example, during very cold winter days when temperatures remain well below zero for the whole day, the first and second PQ-phases may overlap without a clearly distinguishable NPQ-phase in between -illustrating the temperature inhibition of NPQ induction (Fig. 6a, e, i). In contrast, during summer days, as well as in most published studies, one can predominantly observe the first two phases (Fig. 6c, g, k) or even just the first PQ-phase, which is common on cloudy days when NPQ activity is minimal (Fig. 6d, h, l). Additionally, the PQ- and NPQ-phases can be strongly dampened (Fig. 6j vs k) under conditions of strong NPQ (Fig. 6f vs j). These examples highlight the informative potential of long-term, high-resolution, and in situ PAM ChlF. Such data can not only help us characterize the environmental regulation of the light reactions of photosynthesis, but support also their modelling and integration into larger modelling schemes (Bacour et al. 2019; Gu et al. 2019; Raczka et al. 2019). For further insights into the underlying physiological mechanisms in the pine and spruce foliage investigated in the present study we recommend the reader to browse through previous studies at the same site (Grebe et al. 2024; Porcar-Castell 2011; Rajewicz et al. 2023; Zhang et al. 2019).

## Final remarks and future steps

PAM ChlF measurements provide a non-invasive and effective means to monitor the environmental regulation of the light reactions of photosynthesis in leaves and in situ. When combined with photosynthetic gas exchange measurements (Yin et al. 2011) -now also feasible in the field over extended periods (Oivukkamäki et al. 2025)-, leaf-level PAM ChlF measurements provide a complementary view of photosynthesis, contributing to a more comprehensive understanding of its complex regulation under natural conditions. Furthermore, the ability of PAM ChlF to resolve the temporal dynamics of photochemical (PQ) and non-photochemical quenching (NPQ) under natural conditions provides unique opportunities to characterize and model their regulation. This is a key step toward mechanistically linking photosynthesis to the various optical signals generated by the dynamic regulation of the light reactions -signals now measurable at multiple scales through remote sensing methods, such as the emission of solar-induced fluorescence (SIF) (Porcar-Castell et al. 2021; Sun et al. 2023a,b)- offering a means to study photosynthesis at scales inaccessible to other methods.

In this communication, we presented a set of guidelines, recommendations and data processing tools to support current and future users in successfully implementing long-term field PAM measurements. We also introduce and demonstrate a theoretical framework for the mechanistic interpretation of such data, both in terms of physiological regulatory processes (PQ and NPQ), and of potential caveats associated with departures from the underlying assumptions.

The processing code filtered out most of the spurious observations due to meteorological factors like condensation, rain, snowfall or ice formation, which affect *PAR*_*ML*_ and *β* in Eqs. 1and 2. In addition, changes in leaf PAR absorption (*A*), the partitioning of excitation energy between PSI and PSII (*α*_*II*_), and the contribution of PSI fluorescence to the measured signals (*C*_*PSI*_) (Pfündel et al. 2013) can all lead to deviations from Assumption 1, requiring an adjustment in the reference ${F_{MR}}$, or an alternative means of correcting PAM-F levels before calculating PQ and NPQ yields and parameters. This is a critical step since departures from Assumption 1, for example a decrease in leaf absorption, can be wrongly interpreted as an increase in NPQ if not accounted for. Unfortunately, although these factors can vary significantly during the seasons, practical methods and protocols to record their variation in the field are still lacking. For example, leaf and needle PAR absorption measurements typically require the use of integrating spheres (Olascoaga et al. 2016), whereas estimation of *a*_*II*_ and *C*_*PSI*_ typically involve comprehensive measurements of PAM-F, gas exchange and spectrally resolved ChlF (Genty et al. 1990; Laisk and Loreto 1996). These approaches are still awaiting to be adapted for routine field monitoring. In the future, integrated measurement protocols that combine field PAM-F, gas exchange, and spectral data could provide valuable insights into these dynamics, enhancing our understanding of the mechanistic regulation of photosynthesis and improving the interpretation of PAM-F data. Integrated measurements of long-term PAM ChlF can also provide novel information to develop and validate next generation of quantitative, process-based and mechanistic models of photosynthesis (Porcar-Castell et al. 2021; Gu et al. 2019; Han et al. 2022; Sun et al. 2023a,b). Until then, users should be aware of the potential impact of changes in these factors when interpreting long-term PAM-F data.

Lastly, recent studies have challenged the validity of Assumption 2 (i.e., PQ = 0 during a saturating pulse), either due to sub-saturation in the presence of strong NPQ (Loriaux et al. 2013), or to the presence of multiple fluorescent states in the closed PSII reaction centres (Garab et al. 2023). Furthermore, the presence of variable fluorescence in PSI remains also under debate (Pfündel 2021; Schreiber and Klughammer 2021). The impact of these phenomena on the interpretation of long-term PAM-F will still require careful assessment. On a positive note, some manufacturers are beginning to implement corrections to address these issues. For example, by incorporating the multiphase method presented by Loriaux et al. (2013) to address the potential sub-saturation of SP under high NPQ conditions. Enhanced collaboration between scientists and industry will certainly continue to contribute to the advancement of PAM ChlF methods.

## Electronic supplementary material

Below is the link to the electronic supplementary material.

Supplementary Material 1

## Data Availability

The link to the R-package and to the datasets is provided in the manuscript and available via Github.

## Code availability

https://github.com/chaoxzhang/LongTermPAM.
