# Supplementary material for: A practical guide to long-term field PAM chlorophyll fluorescence measurements: setup, installation, data processing with R package ‘LongTermPAM’ and interpretation
Source: Photosynth Res. 2025 Sep 4;163(5):45. doi: 10.1007/s11120-025-01166-1 (PMC12411321; doi:10.1007/s11120-025-01166-1)
Supplement: Supplementary file 1 — Supplementary Material 1 [file 11120_2025_1166_MOESM1_ESM.docx]

**Supplementary Information to “A practical guide to long-term field PAM fluorescence measurements: setup, installation, data processing with R package ‘LongTermPAM’ and interpretation**

Chao Zhang^1^, Erhard E. Pfündel^2^, Jon Atherton^1^, Juho Aalto^3,4^, Jia Bai^1,5,6^, Toivo Pohja^4^, Paulina Rajewicz^1^, Albert Porcar-Castell^1,*^

**Supplement 1. Correcting the temperature dependence of PAM-ChlF measurements**

To correct for the temperature dependence of F’ and F_M_’ estimation, users can apply a standard correction function supplied by the manufacturer or prepare a specific one. In the case of the Monitoring PAM system from Walz, we have identified a temperature dependence of the ChlF-signal both when a fluorescence standard is placed in the leaf clip and when the clip is empty. This suggests that the temperature dependence of the ChlF signal includes two components: (1) a sample-independent component (i.e., independent of the ChlF level of the sample), described by the temperature response of the empty clip and referred to here as the “temperature dependence of the offset”, and (2) a sample-dependent component (i.e., the increase or decrease in ChlF signal is proportional to the level of ChlF emitted by the sample), which is here referred to as “the temperature dependence of the signal”.

The correction function can subsequently be estimated with (two-factor correction) or without (one-factor correction) separate consideration of both processes to the offset. Since the temperature dependence of the F’ and F_M_’ estimation is slightly different, users will require separate correction functions for F’ and F_M_’. For the one-factor correction (where the temperature dependence of the offset and signal components are combined), the overall temperature dependence of the measuring heads can be estimated by placing a fluorescence standard into the clip and measuring F’ and F_M_’ across a meaningful range of temperatures using a temperature or climate chamber. The results can then be normalized to a standard temperature, and a regression function can be fitted to capture the relative changes in F’ and F_M_’ as a function of ambient temperature. These regression functions can be subsequently used to correct the measured F’ and F_M_’ levels. For a two-factor correction, the dependence is estimated in two steps. First, the temperature dependence of the measurement offset is determined by recording the temperature dependence of F’ and F_M_’ with an empty clip. Second, the combined temperature dependence of the signal and offset is determined using the fluorescence standard in the same manner as described above for the one-factor correction. The temperature dependence of the pure signal component can be then obtained by subtracting the previously measured offset dependence. A two-factor correction may be preferable if sample signals are very small, but it will require adjustment if the measuring light (ML) settings are changed, since the offset correction is subtracted in absolute mV units.

It is important to note that the ChlF yield of the standard foils is also temperature dependent. Therefore, for calibration purposes, the standard foil should remain at a constant temperature while the head is subjected to temperature changes. This can be achieved by using fibre optics in combination with a climate chamber, so that the standard foil can be measured outside the chamber while the measuring head temperature is varied inside the climate chamber. Alternatively, the temperature dependence can be estimated by cooling the measuring heads in a freezer (e.g., –20 °C) and then quickly connecting them to their leaf clips containing the standard foil at room temperature (e.g., 25 °C). By supplying a saturating pulse every 30-60 seconds, users can record the decrease in F’ and F_M_’ as the measuring head warms up from –20 °C to 25° C, using the internal temperature recording to build the correction function. This method assumes that the temperature reading of the measuring head is accurate, which should be also verified. In conclusion, unless users have access to the necessary facilities, we recommend using a default one-factor calibration provided by the manufacturer, which should be sufficient under most conditions.

**Supplement 2. Adjusting the sensitivity of the fluorescence detector**

Advanced users can adjust the sensitivity of the fluorescence detector to cross-reference different measuring heads to a single standard. In the case of the monitoring PAM system from Walz, this can be achieved by placing a fluorescence standard in the clip, adjusting the sensor gain until reaching the desired reference level, and then performing a zero-offset measurement with a non-fluorescent standard or pointing the sensor to a non-fluorescent target. After repeating this process a few times, the measuring head will be eventually aligned to the desired level. Repeating the same procedure with the other heads will bring them all to the same sensitivity level. We recommend checking the signals obtained with the fluorescence standards during each regular maintenance cycle.

**Supplement 3. Calibrating the PAR and temperature sensors**

It is good practice to regularly compare the PAR and temperature readings of your sensors to those from a calibrated reference sensor. In the case of the monitoring PAM system, and also the Plant Stress Probe from Opti-Sciences, the PAR sensors provide only an indirect estimation of the PAR experienced by the leaf. Calibration of the Monitoring PAM PAR sensor requires stable sunny outdoor conditions (preferably >1000 µmol PAR) and an independent, calibrated quantum PAR sensor. To begin, place a dummy leaf sample in the clip to simulate typical measuring conditions and position the sensor plate horizontally ensuring that the measuring head is in the opposite direction to the sun. Users should avoid highly reflective backgrounds. Next, adjust the PAR sensor gain until its reading matches that of the calibrated PAR quantum sensor placed in the same geometry. Then, place the fluorometer in a dark box to adjust the PAR sensor offset. This procedure is then repeated multiple times until both readings are within a few µmol of the calibrated quantum sensor. For more precise co-registration, the calibrated PAR sensor can be mounted in a dummy measuring clip to better simulate actual measurement conditions.

Temperature sensor calibration can be performed similarly but requires a climate chamber and can take days. For the monitoring PAM system from Walz, it is important to run the system in Stand Alone mode, as temperature readings tend to increase by a few degrees when operating in Wincontrol or online mode, -likely due to internal ML LEDs dissipating heat.

**Supplement 4. Field Installation supports and tips for installation of MONI-PAM.**

The design of the Monitoring PAM field installation supports is shown in Fig. S1. These supports are intended for mounting monitoring PAM-type fluorometers (with a cylindrical design) on woody plants with branches capable of bearing some weight. The supports consist of three essential components: the **head holder rings** that secure the measuring head and allow for some adjustment, the **connecting plate** that can be affixed in various configurations and allows the user to adjust the angle of the fluorometer relative to the branch being measured, and the **central plate** that connects these two components together and provides overall flexibility to the installation. In addition, the supports include optional accessories such as the **twig holder,** which can be attached in different positions on the central plate to provide additional support to the twig, and the **line holder**, which can help bear part of the system’s weight when mounted horizontally, particularly in the intermediate sections of a tree canopy. One last essential piece (not shown in the diagram) is a **metal bar** including two screw holes to attach the connecting plate and provide a means to secure the supports to the tree branch. The length of the bar can be selected depending on the mounting position (typically between 0.5 and 1.5 m). The STEP files for the manufacture of each component can be downloaded from Zenodo (https://doi.org/10.5281/zenodo.14961722).

Other essential installation accessories include cable ties and soft foam paddings, which should be placed wherever metal parts are in contact with the branch to minimize frictional damage. Narrow strips of soft foam are also recommended for the leaf clip to provide a softer contact with the leaf. Cable ties of various sizes can be then used to secure the branch to the bar and to fix the measured twig to the central plate or leaf clip. It is also recommended to install the measuring heads so that they point downward at an angle of at least 20°. Additionally, for installations in the Northern hemisphere, the optical end of the sensor should ideally face south to minimize self-shading.

**Supplement 5. Data processing with LongTermPAM R-package**

The data processing includes three phases (Fig. 2):

**Data Preparation**

***readPAM() function***

The function *readPAM()* reads, organizes and combines all the MONI-PAM data from an observation period into a single file. In addition, because sunlight is a key factor affecting the regulatory dynamics of F’ and F_M_’, it becomes practical to conduct certain data filtering steps within specific illumination periods, such as nighttime (i.e., period between dusk to dawn), or morning (defined as period between dawn to solar noon). For this purpose, we use getSunlightTimes() function from suncalc R-package to retrieve dawn, sunrise, solar noon, sunset, and dusk and add this information in the re-organized dataset. Examples of an original and re-organized MONI-PAM files are shown in Fig. S2. It is also recommended to remove the first 2-3 days of observations from the long-term analysis, because leaves need a few days to acclimate to the new light environment after being installed in the clip with a new orientation.

***Correct() function***

This function applies a two-factor correction to correct for the temperature dependence of the LED during the F’ and F_M_’ (equations S1 and S2). See Supplement 1 for details.

| ${F'}_{corrected}= \frac{{F'}_{measured}-(-0.417 \times T\left( ℃ \right)+8.15)}{-0.00265 \times T\left( ℃ \right)+1.058}$ | eqn S1 |
| --- | --- |
| $F_{Mcorrected}^{'}= \frac{F_{Mmeasured}^{'}-(-0.447 \times T\left( ℃ \right)+22.62)}{-0.00224 \times T\left( ℃ \right)+1.046}$ | eqn S2 |

**Data Filtering**

The R Package includes six separate R functions to identify and remove spurious PAM-F observations (Fig. S8). The functions compare the pattern of variation in F’, F_M_’ and Y(II) (i.e., ΦP) during nighttime, morning hours, as well as between different nights. Spurious cases are identified by calculating and comparing the percentage change of F’, F_M_’, and Y(II) between consecutive time points. Different percentage change thresholds are applied for filter functions 2-5. Some of these thresholds have a fixed value, whereas others can be adjusted by the user to fine tune the filtering process (Table S1). Initial suggestions to define the key thresholds are shown in Table S1. Further details on how to define the threshold parameters are described in the documentation of the package.

***filter1.lowF() function***

PAM-F levels can drastically decrease when snow, ice or water appears between sample and sensor. The *filter1.lowF()* function is built to filter and remove extremely low F’ and F_M_’ values when either F’ was below 10 or F_M_’ was below 50. We did not set a higher threshold here, because the other five filter functions will provide better solutions to filter other relatively low and unreasonable F’ and F_M_’. Another special case in which Y(II) was not automatically recorded was when F_M_’ ≤ F’. In such case, *filter1.lowF()* will also remove the corresponding F’, and F_M_’, if F_M_’ < 50 but it will retain the values if F_M_’>50 to avoid the systematic removal of points where Y(II) approximates zero, when measurement noise could cause F’ to be higher than F_M_’.

***filter2.night()*** *and* ***filter3.day()*** *functions*

We use *filter2.night()* and *filter3.day()* functions to identify and remove spurious data resulting from water condensation, frost or other unknown reasons. These can occur either during nighttime or daytime, but the underlying logic is slightly different since patterns that may be flagged as abnormal during nighttime can be common during daytime. For example, a sudden decrease in F_M_’ with minor effect on Y(II) would be a clear sign of spurious data during nighttime but this is not so clear during daytime when these fluctuations in F_M_’ and Y(II) respond also to changes in illumination and temperature, calling for two separate functions.

***filter4.FVFM()*** *function*

We use *filter4.FVFM()* function to identify and remove spurious F_V_/F_M_ observations (i.e., maximum daily Y(II) or ΦP). F_V_/F_M_ is, by definition, obtained during nighttime (from sunset to sunrise) only. First F_V_/F_M_ is defined as the maximum Y(II) obtained for that night after filters 2 and 3 above. This filter function then compares the day-to-day variability in F_V_/F_M_ along that of F_M_ (e.g., day 1 vs day 2). Typically, F_V_/F_M_ increases or decreases gradually from day to day along with F_M_, in contrast, disturbances like snow, ice or persistent rainfall or condensation can drastically decrease F_V_/F_M_ between days with only a minor impact on F_V_/F_M_. This function will detect and remove these spurious nighttime F_V_/F_M_ values and corresponding F’ and F_M_’. Eventually, when the disturbance source has disappeared and the signals get back to normal, the filter deactivates.

***filter5.expand*()** *function*

Some spurious data points may still remain undetected after applying the previous functions, especially filters 2 and 3 which only focus on a short period of 1-2 days, for example with spurious observations due to snow, frost, or condensation, still remaining at the transition between periods filtered by filters 2 and 3. To address this, in *filter5.expand()* function, we apply a similar filtering logic with *filter2.night()* and *filter3.day()*, but with the filtering window being expanded and dynamic rather than fixed.

**Table S1.** **Key arguments in filter functions and adjustment suggestions.** Higher values for each argument generally result in fewer points being removed. The default value for argument “FmYII” is suitable for this study site. We recommend paying special attention to setting the argument relating to Fm’ threshold (i.e., f2.Fm, f3.Fm, f4.Fm and f5.Fm).

| **Filter function** | **Argument** | **Description** | **Default value** | **Adjustment suggestion** | |
| --- | --- | --- | --- | --- | --- |
|  |  |  |  | **Range** | **Interval** |
| filter2.night() | f2.Fm | Percentage change of Fm’ between consecutive points | 0.03 | 0.01 to 0.1 | 0.01 |
|  | f2.FmYII | Ratio between percentage change in Fm’ and in Y(II) | 3 | 2 to 5 | 1 |
| filter3.day() | f3.Fm | Percentage change of Fm’ between consecutive points | 0.1 | 0.05 to 0.3 | 0.05 |
|  | f3.YII | Percentage change of Y(II) between consecutive points | 0.02 | 0.01 to 0.1 | 0.01 |
|  | f3.FmYII | Ratio between percentage change in Fm’ and in Y(II) | 3 | 2 to 5 | 1 |
| filter4.FVFM | f4.Fm | Percentage change of Fm’ between consecutive days | 0.15 | 0.05 to 0.3 | 0.05 |
|  | f4.FmYII | Ratio between percentage change in Fm and in Fv/Fm | 3 | 2 to 5 | 1 |
| filter5.expand | f5.Fm | Percentage change of Fm’ between consecutive points | 0.2 | 0.05 to 0.3 | 0.05 |
|  | f5.FmYII | Ratio between percentage change in Fm’ and in Y(II) | 3 | 2 to 5 | 1 |
| filter6.adjacent | expand.time | A time window in minutes to decide how many additional adjacent points to remove. For example, if expand.time is 60 and sampling interval is 30 mins, two adjacent points on each side of the previously filtered data will be removed. | No default value. This argument depends on the sampling interval. | | |

***filter6.adjacent*()** function

Finally, with *filter6.adjacent*() the user has an option to remove a variable period or number of additional datapoints prior/ posterior to the section that has been filtered to ensure no intermediate cases remained in the data.

**Parameter estimation**

***ChlFRef() function***

Reference maximal (*F*_MR_) and minimal (*F*_0R_) fluorescence levels are required to estimate ChlF parameters (Table 1). By definition, *F*_0R_ and *F*_MR_ are registered in the absence of NPQ and photoinhibition, when the reference maximum *F*_V_/*F*_M_ tends to be around 0.82-0.84, typically during summertime. To estimate *F*_0R_ and *F*_MR_ in our dataset, we first identify and select a maximum *F*_V_/*F*_M_ and *F*_M_ level for every night using *FindFVFM()* function. If the dataset includes reference (*F*_V_/*F*_M_)_R_ values of 0.82-0.84, those could be directly used to set *F*_0R_ and *F*_MR_. In practice, however, the maximum values of *F*_M_ and *F*_V_/*F*_M_ do not necessarily need to match the same night and, most importantly, it may be that we have a batch of data where *F*_V_/*F*_M_ did not reach the reference values. It therefore becomes practical to build a non-linear regression function between *F*_M_ and *F*_V_/*F*_M_ to obtain *F*_M_R. This function, implemented here with *ChlFRef()*, can be then used to set a user-fixed level for (*F*_V_/*F*_M_)_R_ (set here to 0.83) and subsequently extrapolate the corresponding level of *F*_M_, which then becomes *F*_M_R. Finally, *F*_0R_ can be calculated from (*F*_V_/*F*_M_)_R_ and *F*_MR_ as: *F*_0R_ = [1- (*F*_V_/*F*_M_)_R_]**F*_MR_.

The *ChlFRef()* function can be also used to provide a rapid diagnosis of the filtering process, e.g. to calculate the coefficient of determination (R^2^), and the relative root mean squared error (RRMSE) between (*F*_V_/*F*_M_)_R_ and *F*_M_ before and after data filtering. In addition, this function also includes a visualization of the points, which can provide a rapid way to assess the performance of the filtering, adjust parameters, or consider the need to separate some of the data into separate batches (each with a *F*_MR_ of its own).

***diurnalParams()*** and ***seasonalParams()*** functions

Finally, the package estimates multiple diurnal and seasonal *PQ* and *NPQ* parameters with *diurnalParams()* and *seasonalParams()* functions, respectively, as well as quantum yields of energy partitioning between photochemistry (ΦP, or Y(II) in MONI-PAM data), regulated thermal dissipation (ΦNPQ), and fluorescence and constitutive thermal energy dissipation (ΦF+D) (Table 1).

**Supplementary Figures**


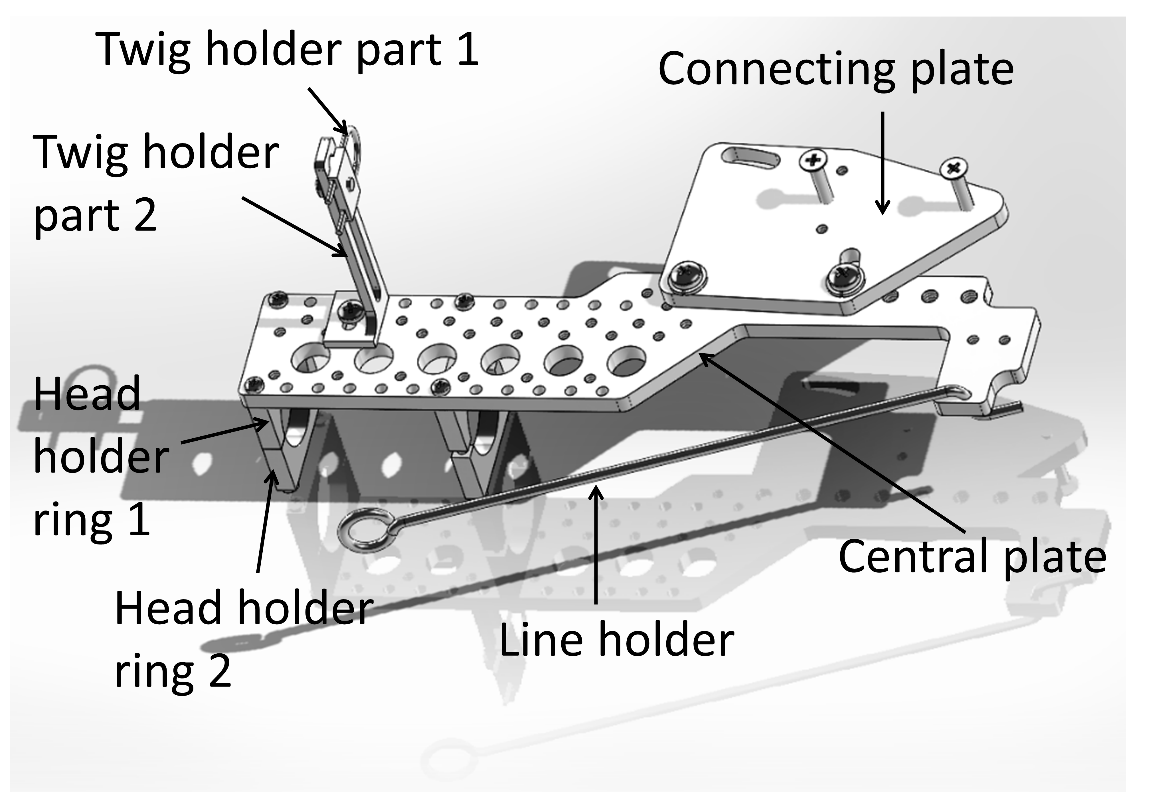


**Fig. S1**. **Main components of the Monitoring PAM field mounting support.** See Supplement 4 for a link to the STEP files to manufacture them.

| **a** | 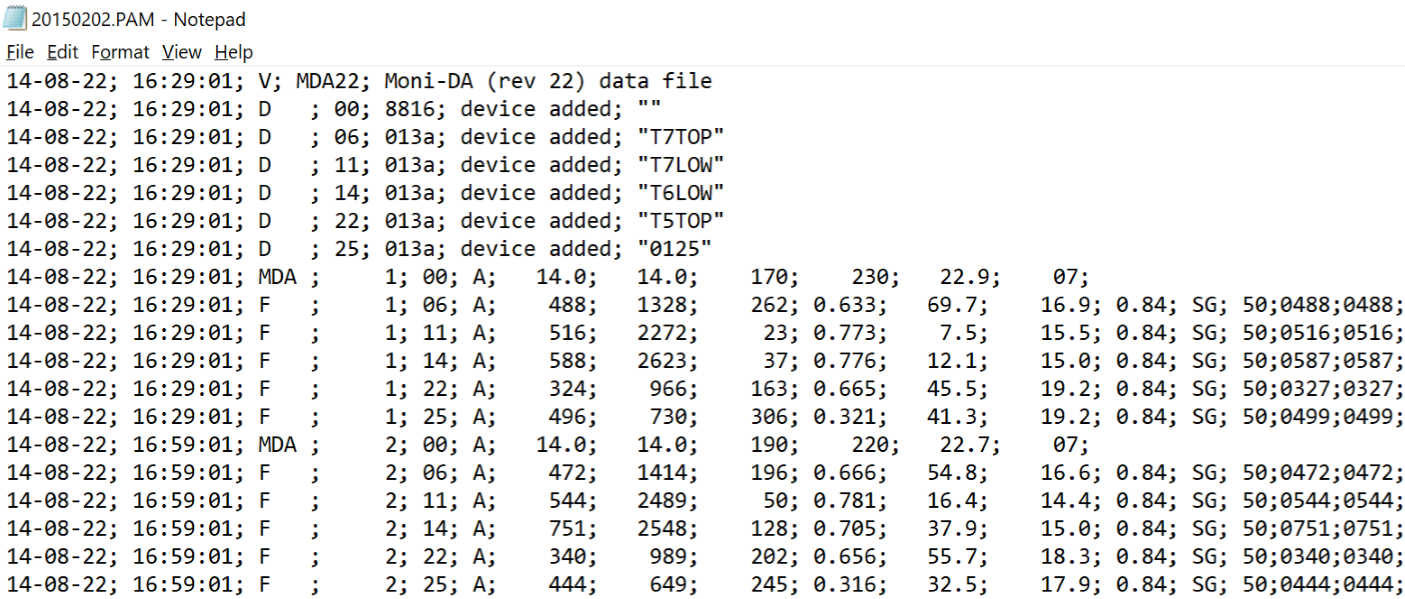 |
| --- | --- |
| **b** | 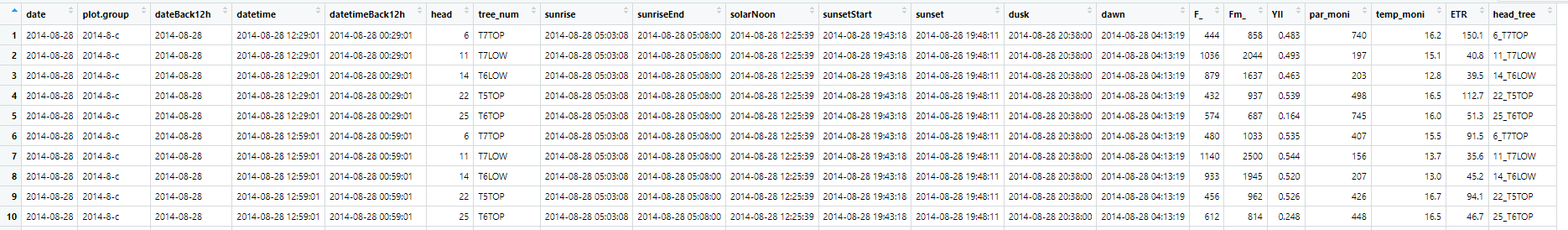 |

**Fig. S2**. **An example of MONI-PAM file structure** opening using Notepad (a) and an example of re-organized MONI-PAM data after applying readPAM() function and opening in R (b). The recorded data in (a) were collected from Aug 22^nd^ of 2014 (first row of first column) to Feb 2^nd^ of 2015 (i.e., file name in the top of the (a), 20150202.PAM

| 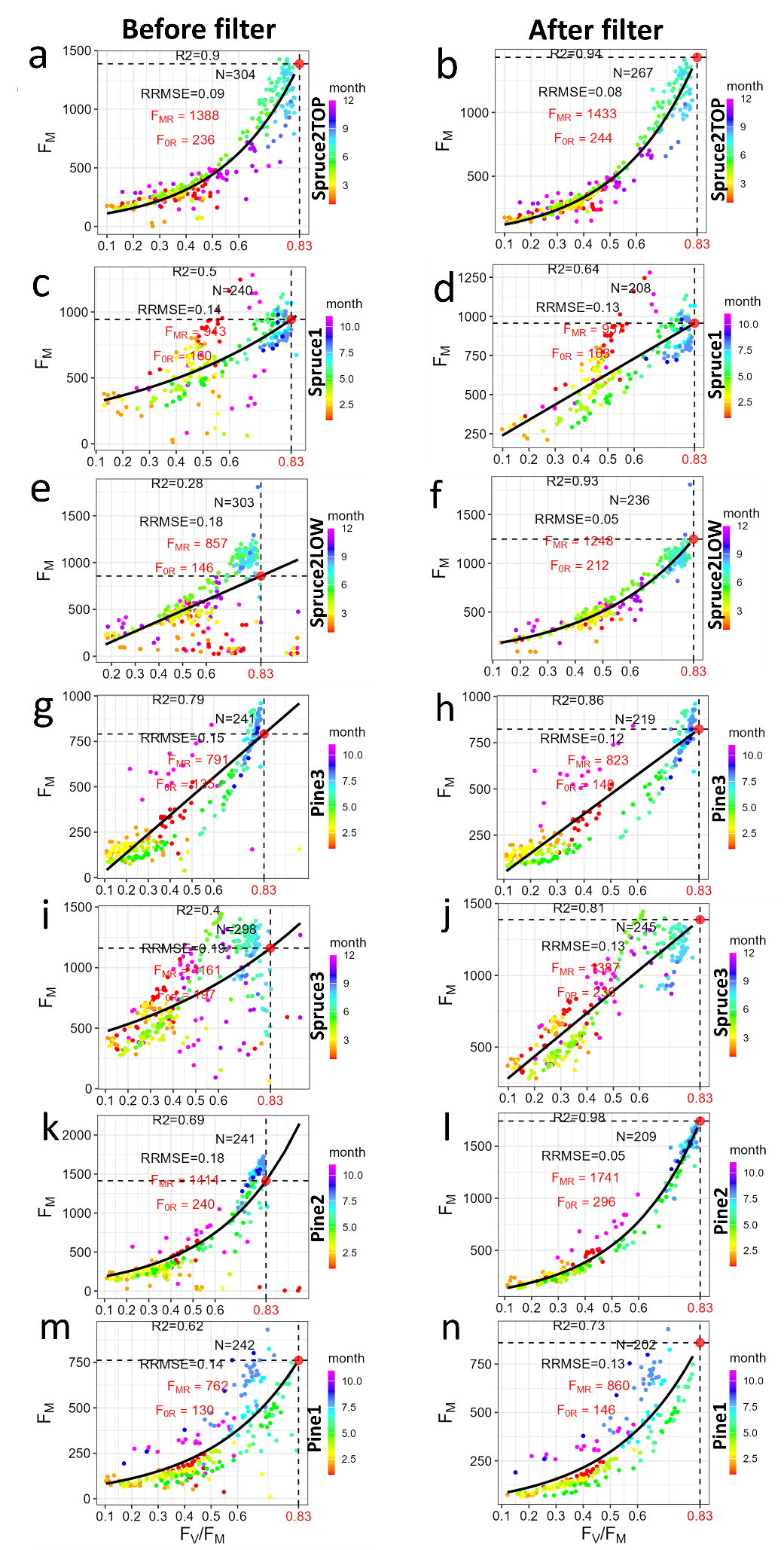 | **Fig. S3**. **Correlations between *F*_M_ and *F*_V_/*F*_M_ before (left column) and after (right column) data filtering** for the 2016-2017 dataset collected in Scots pine and Norway spruce needles. Diagnostic statistics are derived from either a linear or non-linear regression model, including R^2^- correlation coefficient, N-number of points used to fit the model, RRMSE – relative root mean square error. The vertical dashed line indicates the selected reference *F*_V_/*F*_M_ ((*F*_V_/*F*_M_)_R_=0.83) and the horizontal dashed line the corresponding F_MR_ automatically estimated from the fitted model. |
| --- | --- |

**
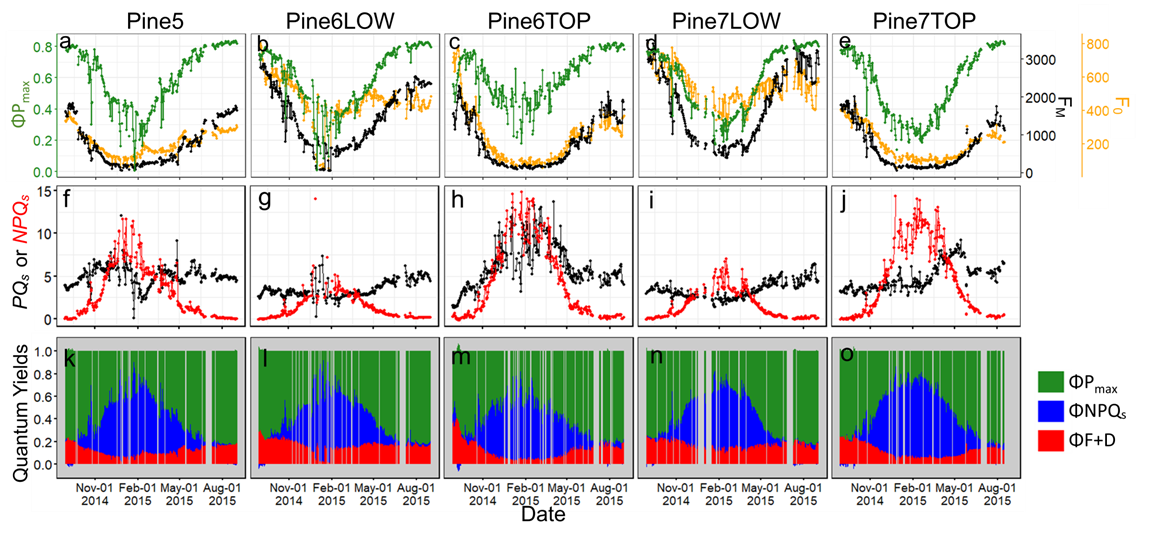
**

**Fig. S4.** **Seasonal variations in ChlF parameters for the 2014-2015 dataset**. Seasonal variation in in $F_{0}$, $F_{M}$ and ${\Phi P}_{max}$ (or $F_{V}$/$F_{M}$) (top panel), *PQ*_s_ and *NPQ*_s_ (middle panel), and ${\Phi P}_{max}$, ${\Phi NPQ}_{s}$, and $\Phi F+D$ (bottom panel).


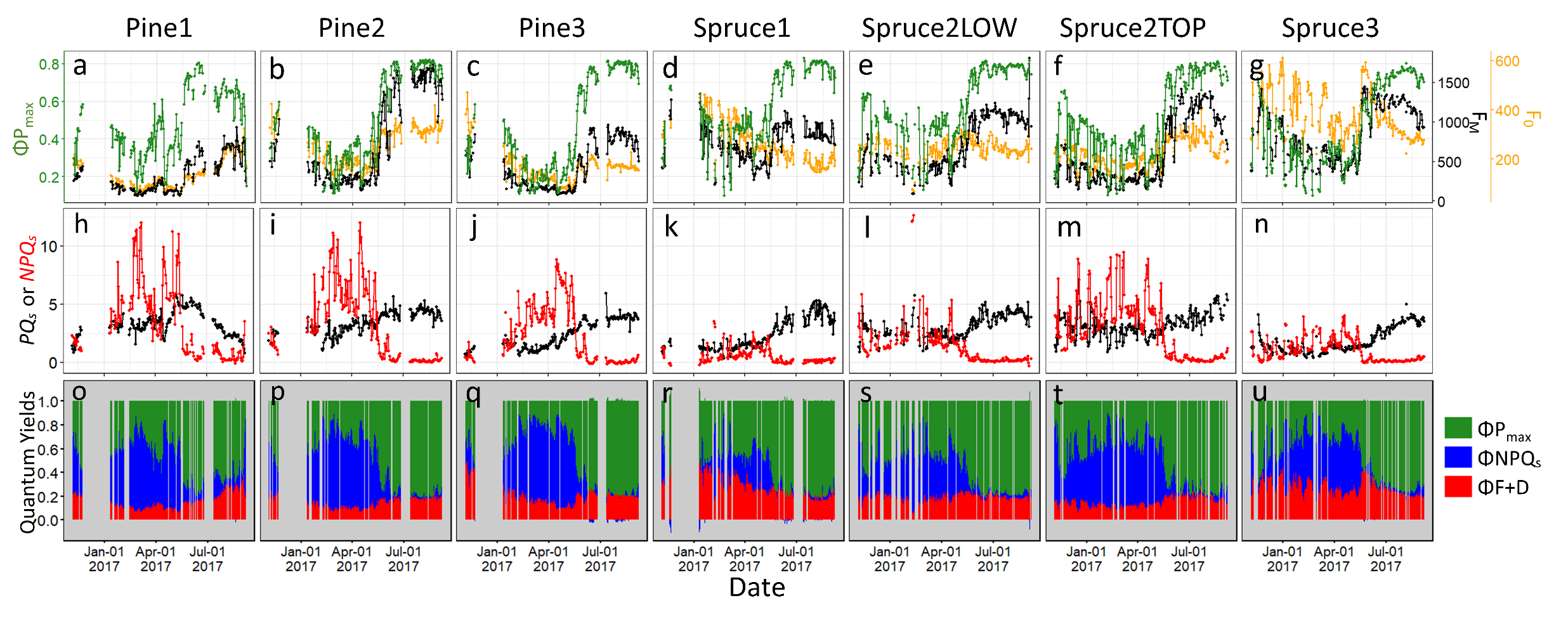


**Fig. S5.** **Seasonal variations in ChlF parameters for the 2016-2017 dataset** Seasonal variation in in $F_{0}$, $F_{M}$ and ${\Phi P}_{max}$ (or $F_{V}$/$F_{M}$) (top panel), *PQ*_s_ and *NPQ*_s_ (middle panel), and ${\Phi P}_{max}$, ${\Phi NPQ}_{s}$, and $\Phi F+D$ (bottom panel).

| 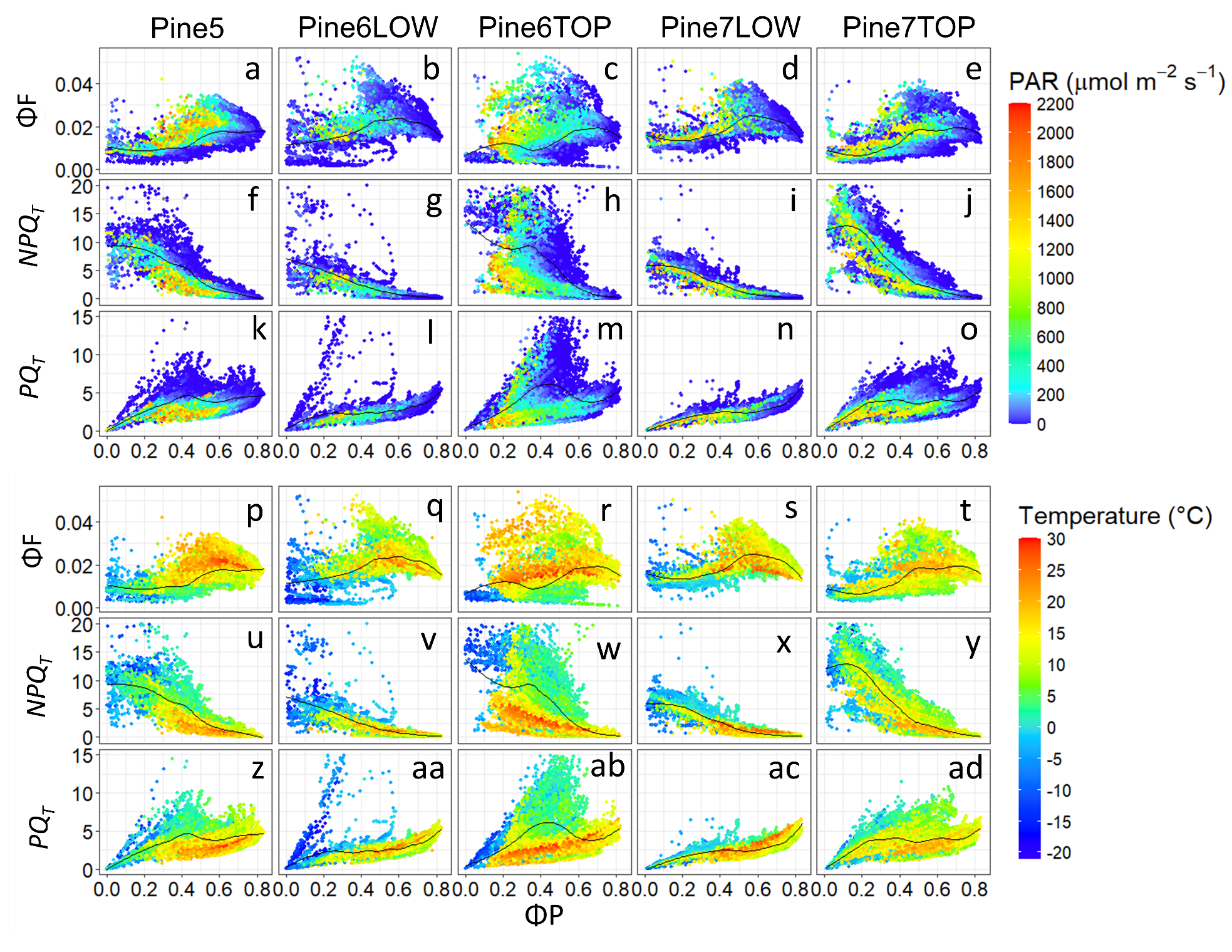 | **Fig. S6**. **Relationships between ChlF parameters and their dependency on PAR and temperature for the 2014-2015 dataset.** Relationships between ΦF vs. ΦP, *NPQ_T_* vs. ΦP, *PQ_T_* vs. ΦP across the whole observation period of 2014-2015 along with changes in PAR (a-o) and temperature (p-ad). We applied generalized additive models (GAM) with integrated smoothness for curve fitting, using the geom_smooth() function from the ggplot2 R package. $\Phi F$ is calculated as 0.1*$\Phi F+D$, assuming a theoretical maximum fluorescence yield in PSII of 10%. |
| --- | --- |
| 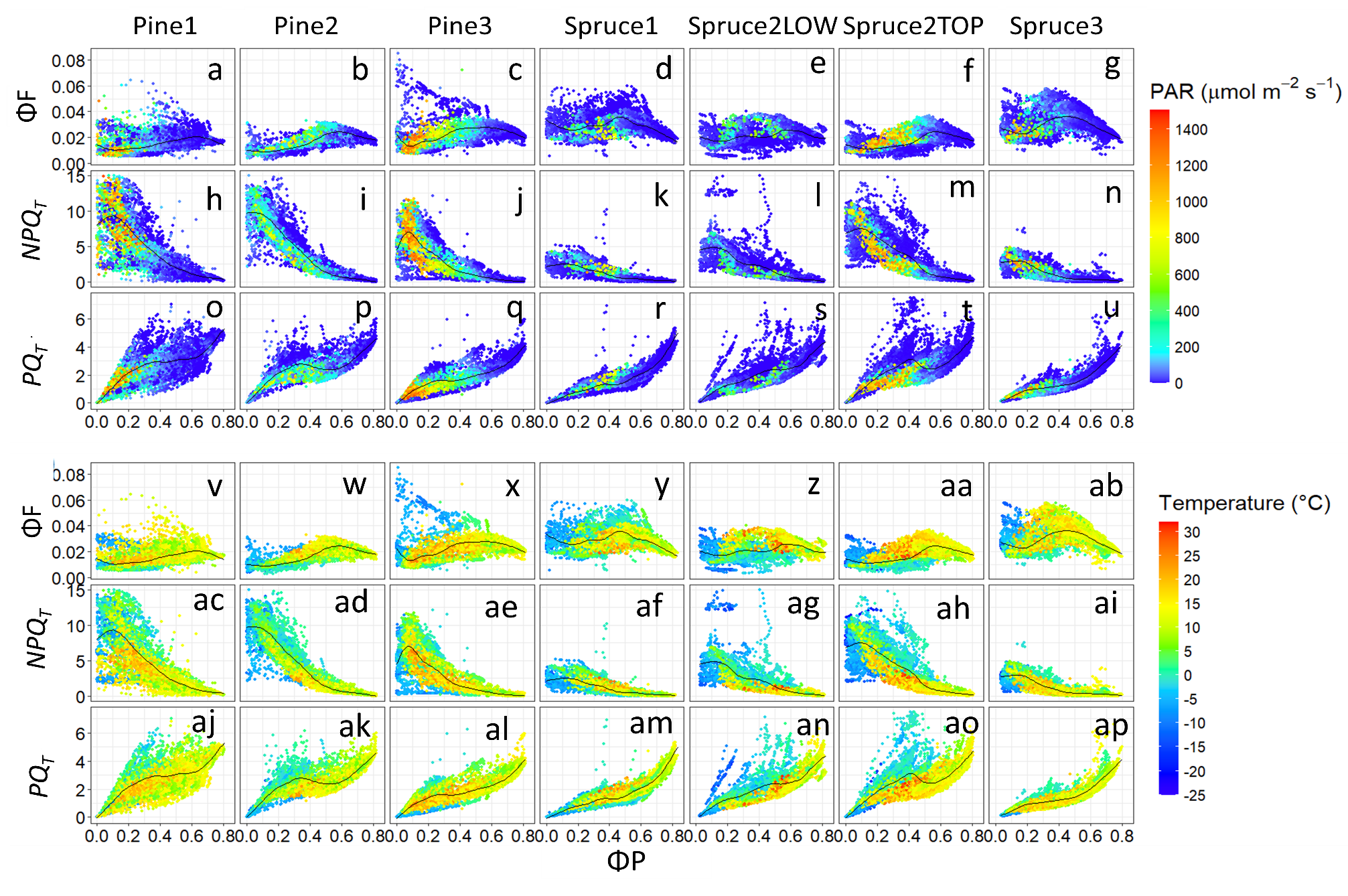  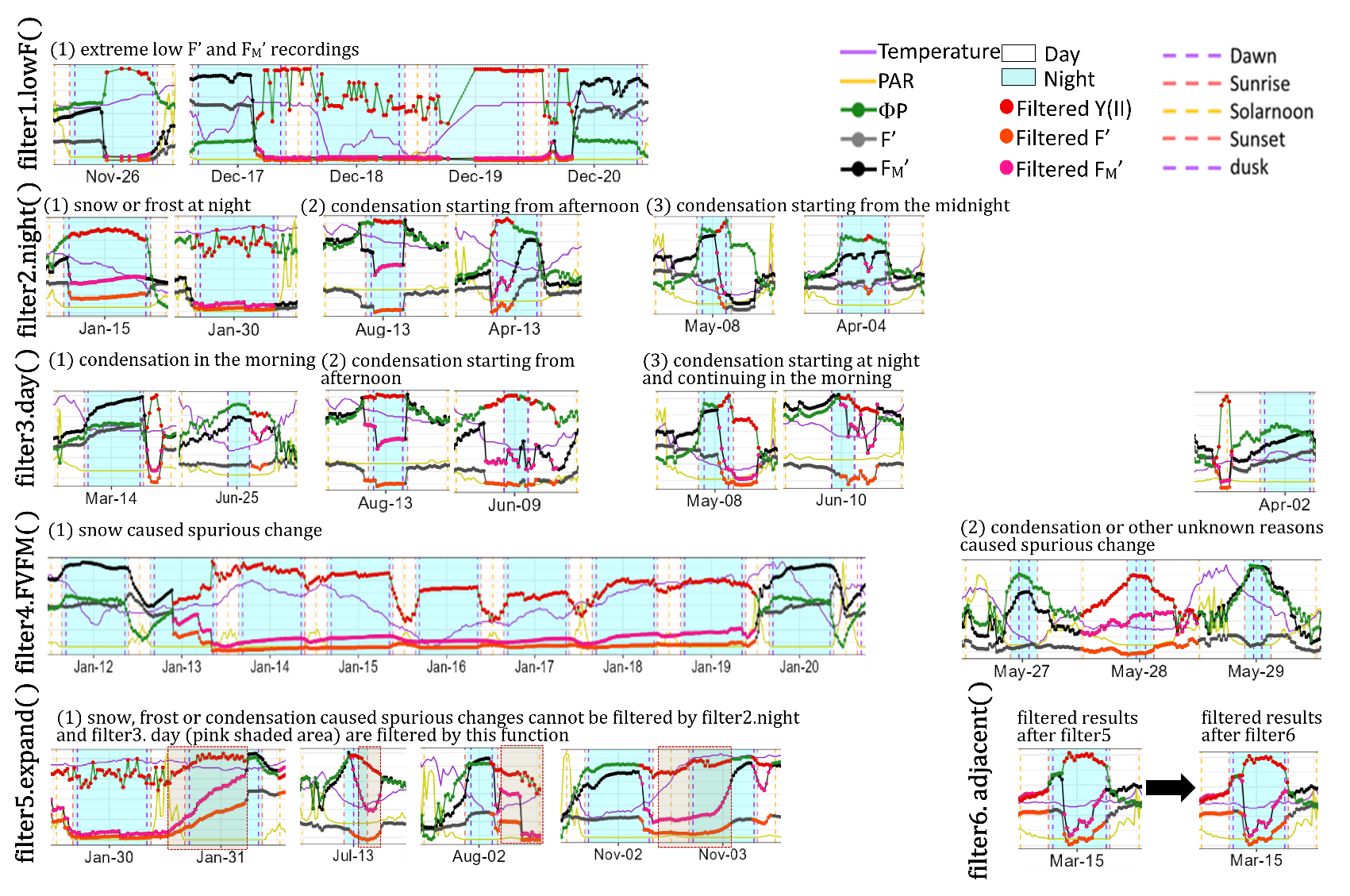  **Fig. S8.** **Examples of filtering steps with LonTermPAM R package**. Green (ΦP or Y(II)), grey (F’), and black (F_M_’) points represent data before filtering, while red (ΦP or Y(II)), orange (F’), and pink (F_M_’) points represent filtered data by current filter function. In filter5.expand(), pink shaded area represents the filtered data by this function. | **Fig. S7**. **Relationships between ChlF parameters and their dependency on PAR and temperature for the 2016-2017 dataset.** Relationships between ΦF vs. ΦP, *NPQ_T_* vs. ΦP, *PQ_T_* vs. ΦP across the whole observation period of 2014-2015 along with changes in PAR (a-u) and temperature (v-ap). We applied generalized additive models (GAM) with integrated smoothness for curve fitting, using the geom_smooth() function from the ggplot2 R package. $\Phi F$ is calculated as 0.1*$\Phi F+D$, assuming a theoretical maximum fluorescence yield in PSII of 10%. |
